# Anatolian genetic ancestry in North Lebanese populations

**DOI:** 10.1038/s41598-024-66191-x

**Published:** 2024-07-05

**Authors:** Daniel E. Platt, Andreas Henschel, Nassim Nicholas Taleb, Pierre Zalloua

**Affiliations:** 1 IBM T. J. Watson Resarch Center, 1101 Kitchawan Rd, Yorktown Hgts, 10598 USA; 2 Center for Cyber-physical Systems, Khalifa University, Abu Dhabi, 127788 UAE; 3 Department of Computer Science, Khalifa University, Abu Dhabi, 127788 UAE; 4 Risk Engineering, New York University, New York, USA; 5 College of Medicine and Health Sciences, Khalifa University, Abu Dhabi, 127788 UAE; 6 Harvard T.H. Chan School of Public Health, Harvard University, Boston, 610101 USA

**Keywords:** Population, Genomics, F4 statistics, Bioinformatics, Genome, Population genetics, Genetics, Molecular biology

## Abstract

Lebanon’s rich history as a cultural crossroad spanning millennia has significantly impacted the genetic composition of its population through successive waves of migration and conquests from surrounding regions. Within modern-day Lebanon, the Koura district stands out with its unique cultural foundations, primarily characterized by a notably high concentration of Greek Orthodox Christians compared to the rest of the country. This study investigates whether the prevalence of Greek Orthodoxy in Koura can be attributed to modern Greek heritage or continuous blending resulting from the ongoing influx of refugees and trade interactions with Greece and Anatolia. We analyzed both ancient and modern DNA data from various populations in the region which could have played a role in shaping the current population of Koura using our own and published data. Our findings indicate that the genetic influence stemming directly from modern Greek immigration into the area appears to be limited. While the historical presence of Greek colonies has left its mark on the region’s past, the distinctive character of Koura seems to have been primarily shaped by cultural and political factors, displaying a stronger genetic connection mostly with Anatolia, with affinity to ancient but not modern Greeks.

## Introduction

The Koura district of modern Lebanon has distinctive cultural institutions likely reflective of its heritage; compared to the rest of Lebanon, it has a high density of, and is almost exclusively, Greek Orthodox Christians. This Lebanese coastal district spans eastward from the Mediterranean coast to the foothills of Mount Lebanon. To the north it abuts Tripoli, the second largest city in Lebanon, and to the south, Batroun, once a major Phoenician port city. It has been the center of olive production for the last several millennia^1,2^. The Koura population has been under sustained cultural influence from the north, mainly Anatolia and this cultural link was maintained throughout its ancient and modern history^3^. Attestation of Anatolian influence was particularly notable under early Egyptian governance of the Levant, and this influence was subsequently maintained and developed through the Byzantine Empire^4^.

Religion has played a major role in the formation of the historic communities in Lebanon. The Koura community is distinctive in its Greek Orthodox and Greek cultural alignments. This raises the intriguing question of whether the population of Koura enjoyed significant immigration of modern Greek Orthodox and/or other northern religious migrants^5^, or acquired its religious and cultural identity by other means. Several prior major events, well documented in historical records, offer suggestions of possible immigration events, or cultural assimilation of Greek customs among non-Greek lineages. For example, following Alexander the Great’s conquests, Greek rulers promoted Greek settlement in the Levant. This left pockets of Greek communities that may have served as “magnets” for Greek refugees during later strife around the Greek homeland. Alternatively, there was substantial cultural and economic affinity between Koura communities and regions in the north that did not directly involve Greece. Therefore, the question of whether or not there is a presence of Greek genetic admixture may provide more details of whether Greek genetic admixture impacted the current population and accounts for the current demic alignment of modern day Koura in north Lebanon^6^.

The Levant went from the Eastern Roman Empire’s grip to the Byzantine Empire which basically replaced the former in 476 CE^7,8^. The Byzantine expansion brought more contact between northern Lebanon and Anatolia. During the Byzantine reign, the Levant and Asia Minor witnessed continuous human mobility primarily due to the many continuing conflicts between the Byzantines and their many rivals, but also for religious pilgrimage^9,10^. While the Romans did not show evidence of genetic mixing with local populations, the Byzantine reign saw many migrations going primarily from west to east^11^. The Byzantine reign was marked by religious councils arguing over heresies coupled with opportunistic political alignments feeding wars to suppress schisms^11,12^. Lebanon offered several regions that attracted refugees of these conflicts. Interestingly, the differentiation was already in place between Koura and populations taking refuge in the near hills driven by the same processes that differentiated Eastern Orthodox from Roman sects^13,14^.

Considering this historical context, we set out to determine whether the genetic makeup of the contemporary population provide insights into the origins of the modern distribution of Greek Orthodoxy. Specifically, whether any genetic impact stems from modern Greek admixture, or from the lasting cultural connection with Anatolia and neighboring northern areas resulting in continuous genetic influx through trade or immigration. In this study, we applied a range of genetic analyses seeking to identify which ancestral populations more closely describe the modern inhabitants of Koura.

This study seeks to elucidate genetic echoes of these geographic foundations that may shed more light on the history of Christianity than simply whether immigration of Greek Orthodox shaped the modern religious populations in Koura. To that end, we sought distinctive alignment between Koura and Byzantine rule, and whether there was substantial admixture with lineages that accompanied Greek Orthodox culture in the genetic record from years 4050 and 1350 before present (1950), prior to the Turkmen arrival in today’s Turkey, the Muslim expansions, the Crusades and the Ottoman rule of the region.

## Results

### Principal component analysis

**Figure 1:**
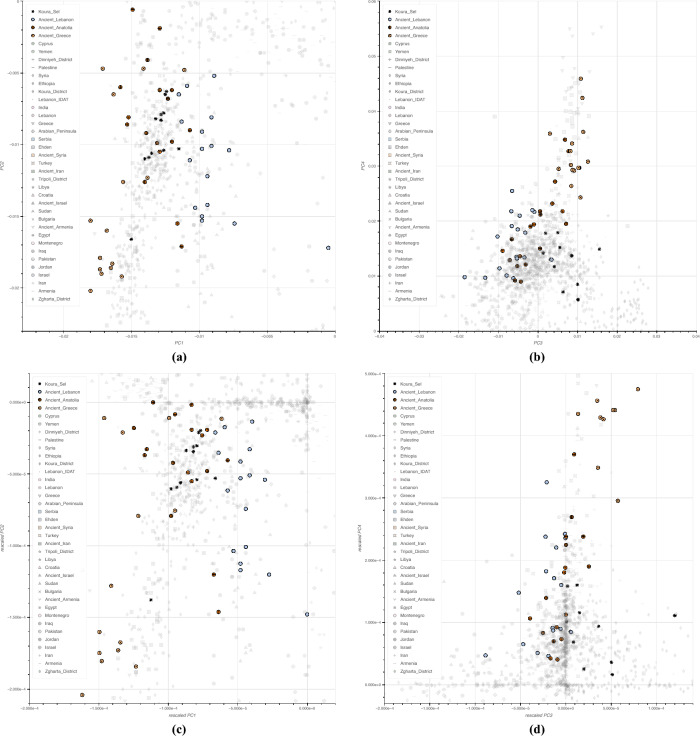
PCA computed and displayed for the full list of populations listed in Supplementary Tables S1 and S2. (**a**) Principal component analysis, PCs 1 and 2. (**b**) Principal component analysis, PCs 3 and 4. (**c**) Rescaled principal component analysis, PCs 1 and 2. (**d**) Rescaled principal component analysis, PCs 3 and 4.

The PCA plot in Fig. 1 revealed several affinities. Ancient Anatolia samples cluster closely to the Koura samples(particularly in PC1 and PC2, but also in PCs 3 and 4), mirroring the results of the admixture plots. While the Koura cluster also significantly overlaps with ancient Syria, it is slightly more distant from the other ancient populations: notably ancient Greeks.

Modern Turkish samples include distinctive differences from the Koura/ancient Anatolia cluster given the intervening admixture of Turkmen. Koura, ancient Anatolia, Cyprus and Dodecanese form a continuum in the PCA of an extended dataset with additional contemporary samples from Greece (with focus on Dodecanese), see Supplementary Fig. S1.

Contemporary Greek PC1 and PC2’s exhibit a relatively wide variance, falling loosely into two main clusters, one more closer to Ehden (a Maronite center) than Koura, the other towards the Asian cline. Contemporary Greeks cluster more distinctly from Koura in the PCA subjected to informational rescaling, as well as in PC3 and PC4 (see Fig. 1).

PCAs restricted to samples of Northern Lebanon communities, shows that Dinniyeh, Ehden/Zgharta and Koura form focal differentiating clusters rather than being randomly mixed (see Supplementary Fig. S2). When ancestral information was considered, while modern Turks show distinctive ancestral patterns among themselves, they do however plot with the remaining Turks away from the Koura samples (Supplementary Fig. 3). Hence, potential geographic/ancestral sample bias is not a concern when comparing modern Turks to the Koura samples. High resolution interactive plots for the above mentioned PCAs are available under https://henschellab.github.io/kourapca/.

### Admixture analysis

**Figure 2:**
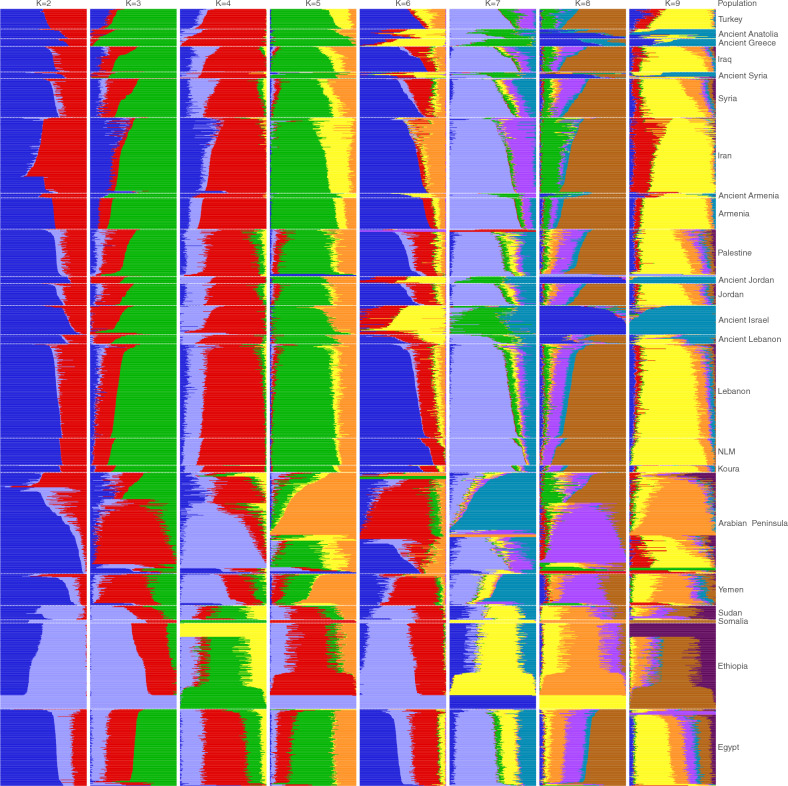
ADMIXTURE plot for admixtures computed on Koura and surrounding populations listed in Supplementary Tables S1 and S2.

ADMIXTURE analyses for $K=2$ to $K=9$ is displayed in Fig. 2. Koura resembles modern Levantine populations, with heavy representation of an ancestral population marked by yellow in $K=9$. This was uncommon in all the ancient populations, except for Lebanon. The cross-validation scores show a broad basin where the unexplained part of most of the variation in the population is near a minimum at K=6 (see Supplementary Fig. S4). Also present in Koura is an ancestral population seen in ancient Armenia, painted red in the $K=9$ analysis, that was rare in the Levant but now much more common. Curiously, ADMIXTURE marked an ancestral ancient Israel component that has become very rare in modern populations.

### F4-statistics and admixture graph

**Figure 3:**
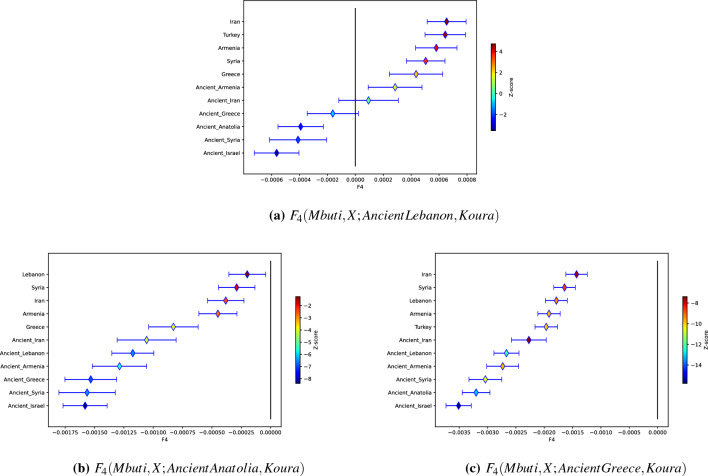
$F_4$ Forest Plots.The $F_4$ plots display the overlap of the differences between Mbuti with population *X* compared to the differences between Ancient Lebanon (**a**), Ancient Anatolia (**b**), Ancient Greece and Koura (**c**).

F4 results are shown in Fig. 3, where the test populations X are the labels of the Y-axis.

In Fig. 3a–c, the $F_4$ output displays the overlap of the differences between Mbuti with population *X* compared to the differences between ancient Lebanon, ancient Anatolia, ancient Greece and Koura. It is worth noting that Koura is a modern population in these analyses. In each case, the ancient population $F_4$s are almost entirely negative. That implies that alleles that emerged between Mbuti and any of those ancient populations are likely shared by alleles that emerged between Koura and all the other ancient populations. In other words, the dominant mutations most likely occurred along lineages that are now heavily represented in Koura. The single exceptions are in ancient Lebanon (Fig. 3a). In this case, ancient Iran had no lineages with significant overlap between Koura and ancient Lebanon, while ancient Armenia shows alleles that are representative of Koura relative to ancient Lebanon. If Koura’s distinctive lineages are older than most of the other populations in the region, Armenia is as old as, if not older than Koura relative to ancient Lebanon. These results generally justify the basic skeleton of the qpGraphs constructed to test the admixture hypotheses we tested between Greece and Koura.

One other feature of the $F_4$ results is that they included modern populations. The modern populations all show significant admixture events differentiating them from their ancient progenitors. Again, the alignment between Koura with ancient Lebanon (Fig. 3a) shows positive alignments between Mbuti and all the modern populations suggesting older lineages similar to those that describe Armenia spread widely since our ancient cutoff. These reflect some of the ancestral populations marked by ADMIXTURE reported above.

The negative F4 values likely indicate a negative overlap on the path from Mbuti to X with the path from ancient Lebanon to Koura, (i.e. alleles accumulated between ancient Lebanon and Koura are found in the alleles accumulated in plotted populations *X* on the path from Mbuti). These results are suggestive of allele sharing between ancient Israel and ancient Lebanon (z = – 3.529), but not with Koura. Koura may not have shared some alleles that ancient Lebanon did share with ancient Israel. Conversely, positive F4 values likely indicate gene flow between X and Koura, but not with ancient Lebanon, the most significant example for X being ancient Iran (z = 4.720), i.e. Koura shares ancient Iranian alleles that ancient Lebanon did not receive.

### qpGraph analysis

**Figure 4:**
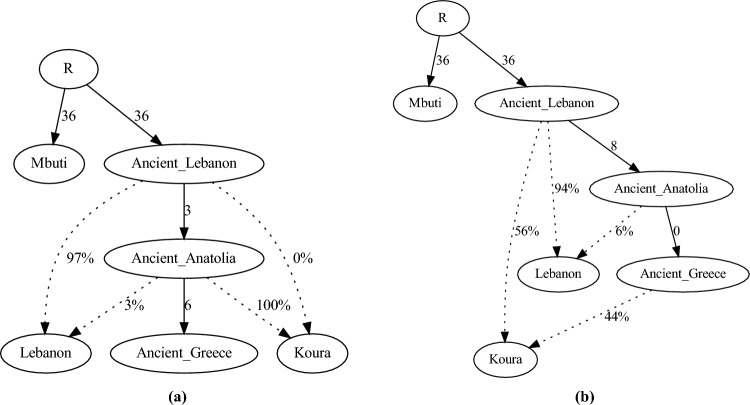
qpGraph topologies. Testing ancient Greek and anatolian contribution to modern Koura Greek orthodox. (**a**) Topology without Greek Admixture - z = − 31.18. (**b**) Topology with Greek Admixture - z = − 41.41.

We designed a basic skeleton to capture relationships between ancient and modern Koura, ancient Greece, ancient Lebanon, ancient Anatolia, and modern Lebanon, to which we could attach candidate configurations to test whether Koura received more Greek or Anatolian genetic contribution to account for Koura’s Greek Orthodox demic character. We also sought to exclude more recent population effects, such as Turkmen admixtures, as possible, by focusing on the ancient populations, seeking lineages reflecting Byzantine reign or earlier Greek admixed lineages. We therefore constructed two topologies, in Fig. 4, which test admixture into Koura with and without Greek contribution. The z-score with Greek admixture was $z = -41.41$, while that without was $z = -31.18$, indicating that the ancient contributions from Greek admixtures explains the modern population more poorly than a model without Greek admixture. The substantial Z scores reveals that a large amount of variation in the modern population is not accounted for by the simple skeleton, but they are adequate to test the amount of variation explained by a candidate Greek admixture.

## Discussion

Koura’s political alignments, recorded through history by Egyptians and other cultures, show affinity with more northern polities. One question is how the modern population of Koura developed such a strong alignment with Greek Orthodoxy, and whether this was accompanied by genetic contribution from refugees or settlers, or whether the alignment emerged due to Koura’s affinity with northern polities, such as Byzantium. We therefore sought to identify lineages marking the regional populations prior to the Turkmen expansions, Muslim expansions, Crusades, and Ottomans, seeking to use those lineages to test for Anatolian and/or Greek admixture into the region.

Despite their geographical proximity, three distinct clusters were observed on a PCA representing samples from North Lebanon. These clusters meet at the center and radiate into three distinct clines representing Koura, and two other Mount Lebanon districts, Zgharta and Dinniyeh respectively, indicating the presence of discrete and specific genetic components differentiating these three geographically close clusters. The Koura population belonging to a coastal region, most probably witnessed human mobility from Anatolia since the Last Glacial Period up until the fall of the Byzantine Empire. Well before the Byzantine rule, the Levant witnessed several successive waves of migration throughout many millennia beginning with pre-Neolithic migrations from the Anatolian plains around 14kya^15,16^. During the Late Bronze Age, Anatolia was home to the Hittites, the Hattians and the Luwians^17^. Western Anatolia was subsequently settled among numerous cultures by the Mycenaean, Ionian and Lidyan Greeks, central Anatolia by the Phrygians and eastern Anatolia by the Urartu and the Assyrians^18^.

The early Anatolian and Iranian farmers also expanded later into the Levant and subsequent human mobility associated with emerging empires and early trade further impacted the genetics of the coastal Levant with recognizable regional affinity^15,16^. Koura constituted an attractive site for the early farmers; it is coastal, with rich farming plains. It is well documented that Koura has been the site of many ancient human settlements, and has been implicated in trade as long as people have traded olives and or cedar wood^19,20^.

Indeed, the Koura samples in the PCA plots fall within a cluster represented primarily by ancient Anatolia, ancient Syria with affinities to ancient but not modern Greeks. These observations persisted even with higher PCA components indicating a close affinity of modern Koura samples with ancient Anatolia. A subset of the Koura samples, who trace back their origin to at least three generations show a very tight cluster at the center of the North Lebanon PCA representing a founding community that was later admixed by neighboring populations.

These results are consistent with the ADMIXTURE plots that resolve at K=6 where cross validation error is minimized (Supplementary Fig. S4). Koura samples and ancient Anatolians show three main components: a large green component representing ancient Greece, a yellow component representing Iran and an orange component representing the Levant and Arabia. The Levant/Arabian component is clearly diminished from the ancient Greeks, while well represented in ancient Anatolia. These admixtures are not surprising given both ancient migrations, Neolithic exchanges, and more modern events such as Persia’s expansion into the Levant founding a navy comprised largely of conscripted Phoenician sailors, and that extended control into Egypt^21^. Their interest in establishing naval ports and ship-building facilities likely made Koura and its surroundings attractive. When Alexander the Great took control of Persia’s Levantine holdings, he left about 1400 of his soldiers behind in the occupation of Phoenicia, and after his death his successor for the Levant, Seleucis, encouraged the settlement of Greeks in the Levant to solidify his grip^22,23^. In fact, the closest individuals to the Koura group on the PCA belonged to the Late Middle Bronze Alalakh population. Alalakh is an ancient settlement around Antioch, the capital of the Seleucid empire. More broadly, the legacy of the Seleucid impact, through the continuation of the satrapy model included colonies that left a legacy of Greek temples and communities; remnants of that program include the temple to Zeus Keraunios at Antioch, and others at Baalbek. This highlights the question of whether Koura was one of these places facilitating subsequent admixture with Byzantines after Roman control due to local cultural affinity, and whether it received substantial Greek settlement. Further, Koura may have appeared to be a safe refuge for Greeks seeking a sanctuary from violence elsewhere due to Koura’s apparent cultural affinity. Therefore, we sought to determine if there was a signal of such settlement. We found that the genetic impact of direct modern Greek immigration appears to be weak in the region. While the established Greek colonies thread their way through the history of Lebanon, the alignment in Koura appears to have been cultural and political, and shows more genetic affinity with Anatolia.

Significant gene flow from ancient Anatolia was also demonstrated using the four-population test (F4) that estimates ancestry proportions. This flow was clearly demonstrated when Mbuti was used as an outgroup (Fig. 3b). These results are suggestive of a continuous gene flow that has been occurring since early movement of people as they expanded from Anatolia into the Levant several millennia ago, and attested to by records from Egyptian period vassal state times onward. Also notable is the significant differences between ancient population samples and modern samples. Differences that maybe attributed to substantial admixture following the fall of the Byzantine Empire that has impacted the distribution of ancestral populations are noted in the ADMIXTURE analysis.

To identify whether the predominate Greek Orthodox population in Koura is due to Anatolian vs. modern Greek admixture, we constructed the qpGraph analysis to isolate pre-Byzantine collapse populations, and test admixture of those components into the modern Koura population. Further, we wished to exclude migrations carried by Turkmen with possible Iranian admixture that came subsequent to Byzantine’s downfall. Greece itself presents a complex history of admixtures reflected in the Language (an early branch of Indo-European) and mythology (displacement of the Titans by the Olympians, and the history of Medusa). Further it was shown that the genetic ancestry of most modern Greeks is still dominated by the Neolithic farmers of western Anatolia and the Aegean and to a lesser extent by the early Iranian and Caucasus farmers^15^.

Greek trade in the Chalcolithic and Bronze Ages competed directly with the Phoenician expansion, and, under Alexander the Great, dominated the world from Greece to India and the whole Eastern Mediterranean. Yet, the genetic interactions of Greek settlements with local populations was complex, and region or even community specific. We note a full analysis of Greece would require a more complete qpGraph skeleton that included elucidation of the Steppes and Indo-European speakers, and then it would be difficult to resolve due to the under-representation of Greek lineages in Koura. The structure within Greece that may be reflected in possible immigration patterns to Koura may be deferred; the question we sought to answer here is whether there was *any* immigration that would have promoted demic Greek Orthodoxy.

We constructed a qpGraph backbone topography based on the ADMIXTURE and F4 results, which indicated a broad-brush flow from Africa towards the north. To that, we added prosthetic admixture candidate configurations to consider the possibility of migration either from ancient Greece or ancient Anatolia. Interestingly, the difference in Z-scores in favor of Anatolia was around 10. This suggests that the presence of Greek culture in the form of Greek Orthodoxy was not due to refugee concentration, or prior Greek affiliation in the Koura region. This also suggests that all the confounder candidates are also absent from the region: apart from the possible Seleucid colonization effects observed in the PCA plots, Alexander the Great’s impacts, and prior Classical era trade, left a relatively small genetic legacy in Koura, a result significant in itself.

Our genetic results appear to recapitulate the long historical record of affiliation with Anatolia. Further, it resolves a question about Seleucid settlement in Koura, and as a possible subsequent magnet for refugee settlement: the genetic signal does not reflect the current demic alignment. Travel to the north and affiliation was easier for Koura than for connections to Lebanon in the south, and those primarily maritime since overland was hard. In sea trade, they were cooperatively affiliated with the Phoenician traders. The other end of the line shows historical ties to Byzantium, which explains the Greek Orthodox affiliation; the historical ties had already been bound over the prior millennia.

## Methods

### Description of samples, populations, and genotyping

In order to compose a comprehensive dataset, we collected genotype array data from various different sources (see supplementary material). 162 ancient DNA samples were drawn from the Allen Ancient DNA Resource (AADR) (https://reich.hms.harvard.edu/allen-ancient-dna-resource-aadr-downloadable-genotypes-present-day-and-ancient-dna-data), version 50.0^24^. Samples were selected from SE Europe and SW Asia between dates 4050 and 1350 before present (1950) to represent lineages from all the populations that may have had an impact on the contemporary population under investigation i.e. comparatively recent ancient populations excluding the Muslim expansion, Turkmen, and other events, plus Mbuti, and are listed in Table S1 with accession labels; publications for the references are listed in Supplementary References. The modern samples collected and analyzed by our team for this study included 1241 samples listed in Table S2. 47 additional samples representing modern Turkey were selected from the AADR (Human Origin dataset, version 54.1^24^). These were selected for the detailed information about their ancestral geographic origins in order to address any potential geographic sample bias for modern Turkey (Table S1).

Note that 318 samples from the modern samples represent the Northern Levant, specifically the districts of Zgharta, Koura and Dinniyeh. From the Koura samples (denoted Koura_District), special scrutiny was given to a selection of 12 samples, denoted Koura_Sel. These subjects were selected based on extended genealogical data availability, placing them in Koura for at least four generations. Seventy samples belonging to two contemporary Greek/Mediterranean datasets^25,26^ from locations geographically close to the Northern Levant were added to perform a PCA with an extended context, see supplementary Table S3. Regarding sample collection from the Northern Levant region, genotype array data was obtained for collected subjects using the Illumina Bead Chips as specified in Table S2, which comprise between 1M to 2.4M typed loci. Genotyping was conducted at the Wellcome Sanger Institute (UK). Modern samples were collected by our team from donors after having given their written informed consent to the project including population analyses. Back-level gene array genotype data were converted to NCBI genome build 37 using LiftOver (http://genome.ucsc.edu/cgi-bin/hgLiftOver).

Plink 1.9^27^ was used to merge all data (including two contemporary Greek/Mediterranean datasets) sources, yielding a dataset with 352K variants. Prior to merging, shared SNPs were selected, polarity was resolved against the aDNA set, tri-alleles were removed.

### Analysis

Principal component analysis was performed using smartPCA^28^ with lsqproject enabled, with numoutevec=10, numoutlieriter=8, outliersigmathresh=6, ldregress=200, ldposlimit=100000, and numoutlierevec=10. We also rescaled the PCA components based on individuals’ genomic mutual information (MI) using the methods described in^29^. Since PCA vectors for Gaussian variables are orthogonal both for correlation and mutual information (MI), rescaling PCA distances using MI, because of its additive properties, allows a representation of relative correlations. This entropy rescaled principal component analysis method, while preserving order relationships, changes the relative distances to make them linear with respect to information, and should reflect time versus number of discriminating SNPs. These results are displayed in Fig. 1.

ADMIXTURE^30^ was applied with default settings over a range of posited ancestral population counts to identify and infer shared lineages from inferred ancestral populations, as shown in Fig. 2. The cross-validation scores are displayed in Fig. S4. The figure was prepared using Pophelper^31^.

F4 analyses were performed exploring $F4 = E[(Mbuti - X)(Ancient\_Lebanon - Koura)]$, $F4 = E[(Mbuti - X)(Ancient\_Anatolia - Koura)]$, and $F4 = E[(Mbuti - X)(Ancient\_Greece - Koura)]$ as shown in Fig. 3.

A qpGraph^32^ scaffold was constructed to test whether migrations from Greece to Koura are substantial compared to a null hypothesis of admixture primarily from nearby regions dominated by admixtures from Anatolian lineages such as from Byzantine Empire. These hypotheses were projected against a shared backbone skeleton representing migrations proceeding from Africa through Levantine populations and continuing north, taking advantage of aDNA , results from ADMIXTURE, and $F_4$ results. The details of the scaffolds and the hypotheses are shown in Fig. 4. Predictivity for candidate topologies with and without Anatolian and Byzantine and Greek admixtures were compared using qpGraph’s z-scores.

Interactive PCA plots were created using the Python library Bokeh^33^.

### Ethical approval

This project was approved by the IRB of the Lebanese American University. All methods and experiments followed IRB approved protocols and were performed in accordance with the relevant guidelines. Participants were informed in writing and verbally before assenting to the study, and a signed informed consent was obtained from every participant.

### Consent to participate

Participants were informed in writing and verbally before assenting to the study, and a signed informed consent was obtained from every participant.

### Supplementary Information

Supplementary Information.

Supplementary Table S1.

Supplementary Table S2.

Supplementary Table S3.

## Data Availability

Data has been submitted to the ArrayExpress collection in BioStudies, Accession number: E-MTAB-13882 and Release date: 2024-03-29.
