# Supplementary material for: Anatolian genetic ancestry in North Lebanese populations
Source: Sci Rep. 2024 Jul 5;14:15518. doi: 10.1038/s41598-024-66191-x (PMC11226446; doi:10.1038/s41598-024-66191-x)
Supplement: Supplementary file 1 — Supplementary Information. [file 41598_2024_66191_MOESM1_ESM.pdf]

Anatolian Genetic Ancestry in North Lebanese Populations - Supplementary Material

Daniel E. Platt[1,+], Andreas Henschel[2,6,+], Nassim Nicholas Taleb[3], and Pierre Zalloua[4, 5, *]

[1]IBM T. J. Watson Resarch Center, 1101 Kitchawan Rd, Yorktown Hgts, 10598, USA
[2]Center for Cyber-physical Systems, Khalifa University, Abu Dhabi, 127788, UAE
[3]Risk Engineering, New York University, New York, USA
[4]College of Medicine and Health Sciences, Khalifa University, Abu Dhabi, 127788, UAE
[5]Harvard T.H. Chan School of Public Health, Harvard University, Boston, 610101, USA
[6]Department of Computer Science, Khalifa University, Abu Dhabi, 127788, UAE
[*]pierre.zalloua@ku.ac.ae
[+]these authors contributed equally to this work

ABSTRACT

Purpose: Lebanon's rich history as a cultural crossroad spanning millennia has significantly impacted the genetic composition of its population through successive waves of migration and conquests from surrounding regions. Within modern-day Lebanon, the Koura district stands out with its unique cultural foundations, primarily characterized by a notably high concentration of Greek Orthodox Christians compared to the rest of the country.
Methods: This study investigates whether the prevalence of Greek Orthodoxy in Koura can be attributed to modern Greek heritage or continuous blending resulting from the ongoing influx of refugees and trade interactions with Greece and Anatolia. We analyzed both ancient and modern DNA data from various populations in the region which could have played a role in shaping the current population of Koura using our own and published data.
Results: Our findings indicate that the genetic influence stemming directly from modern Greek immigration into the area appears to be limited.
Conclusion: While the historical presence of Greek colonies has left its mark on the region's past, the distinctive character of Koura seems to have been primarily shaped by cultural and political factors, displaying a stronger genetic connection mostly with Anatolia, with affinity to ancient but not modern Greeks.

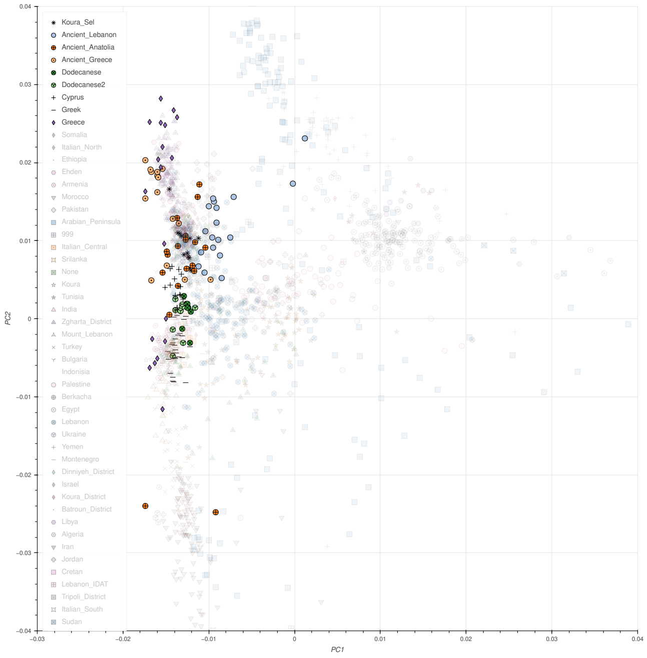

(a) PCs 1 and 2.

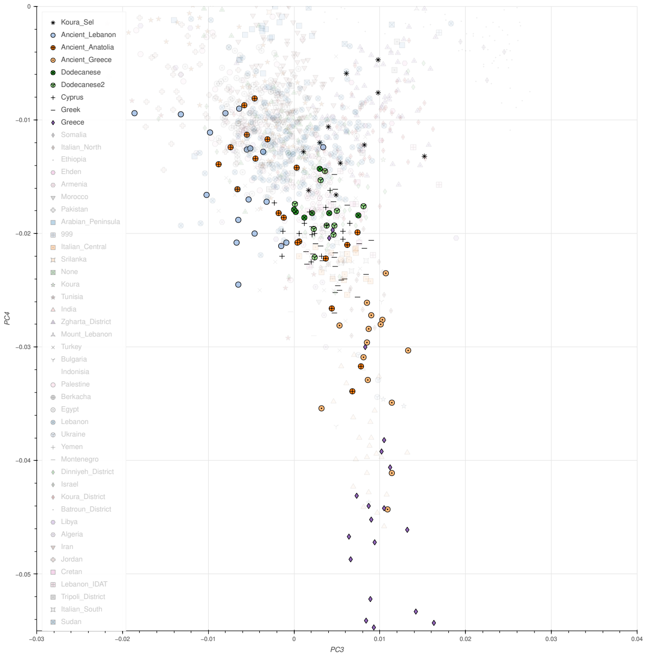

(b) PCs 3 and 4.

Figure S1. PCA with additional Greek/Mediterranean samples as listed in Table S3. Note that Koura_Sel refers to the core set of Koura samples as used in the PCA, admixture, F-statistics and qpGraph analysis shown in Section 2.

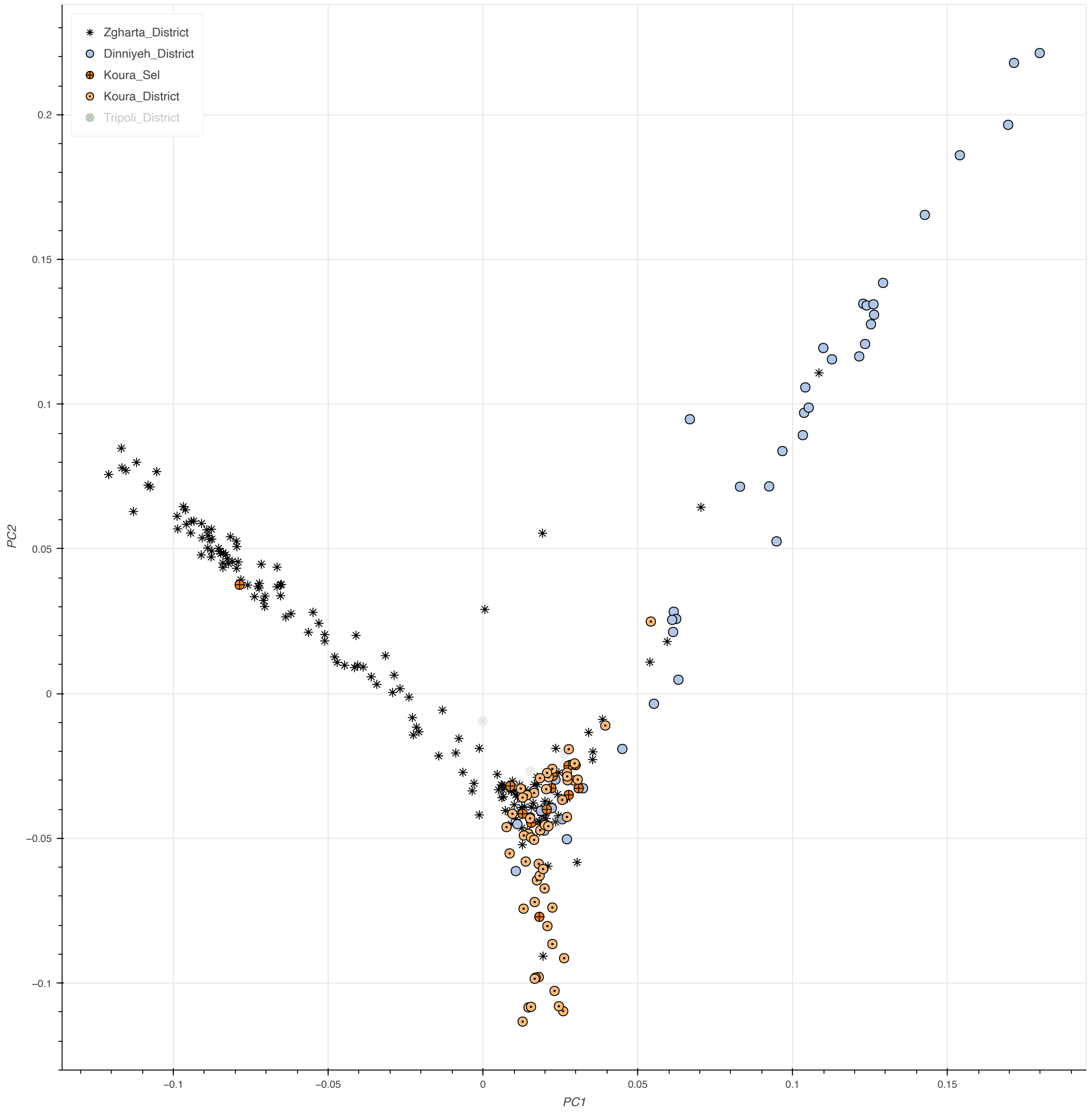

Figure S2. PCA of North Lebanon communities. The respective communities are detailed in Table S3.

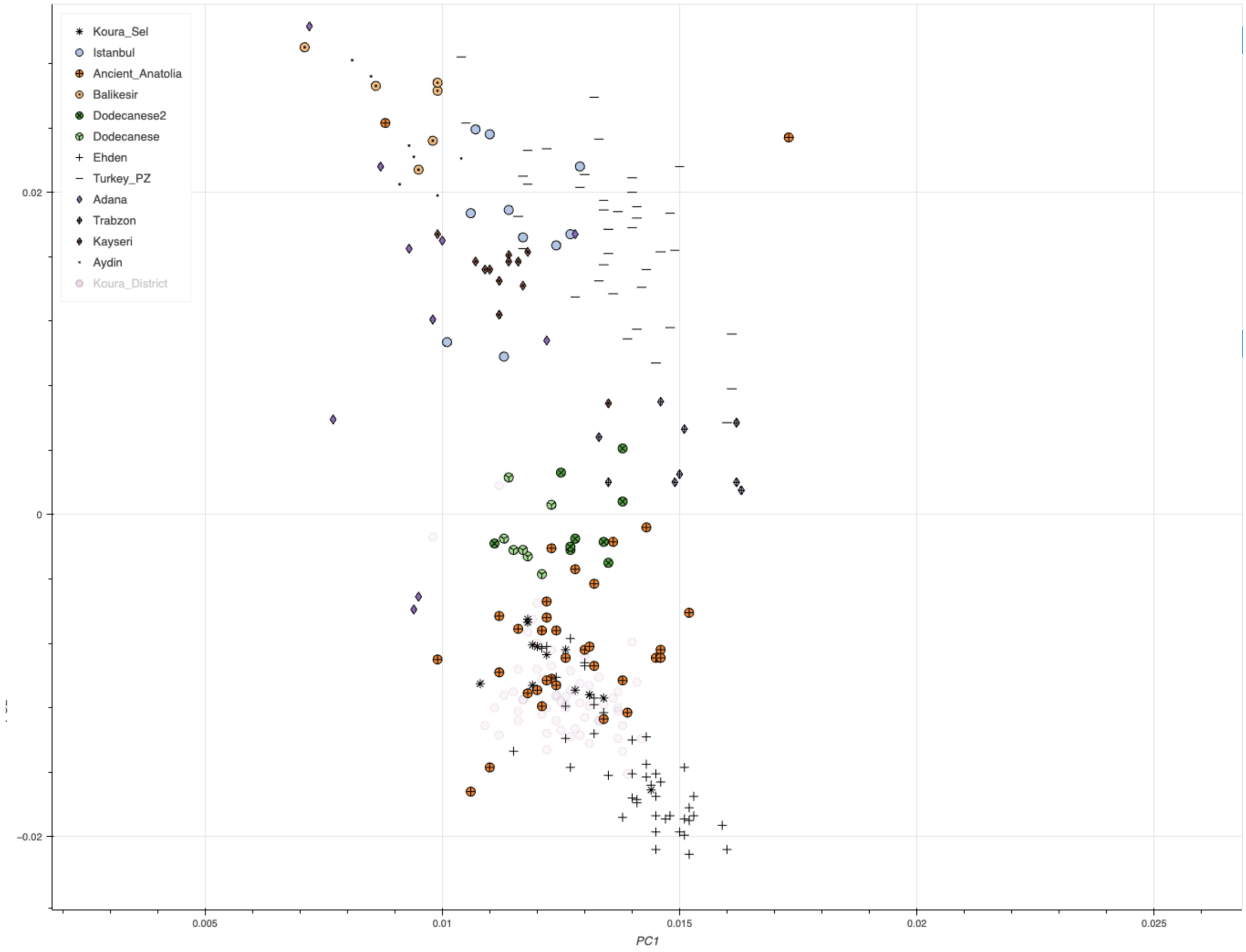

Figure S3. PCA of Koura and modern Turkey populations, which clearly separate.

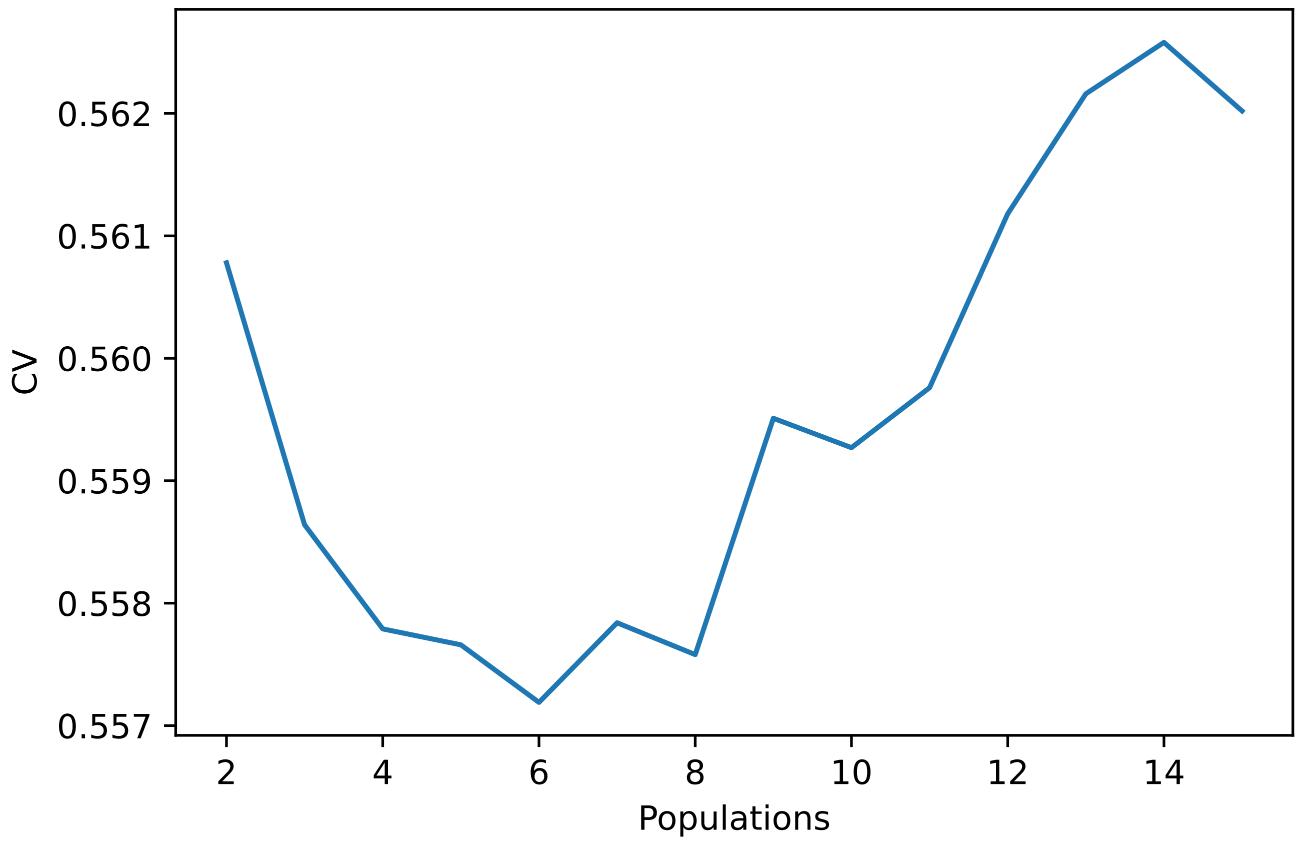

Figure S4. **ADMIXTURE cross validation**

Supplementary Tables Captions

Table S1.Analyzed samples derived from the Allen Ancient DNA. Source populations and dates (before 1950CE) are indicated.

Table S2.Samples collected for the current study. Sample source populations and genotyping platforms are indicated.

Table S3.Analyzed samples from contemporary Greek datasets. Source populations and references are indicated.

Supplementary References

References to genetic datasets utilized via the Reich Lab ancient DNA repository:[1–17]

References

1. Meyer, M. *et al.* A high-coverage genome sequence from an archaic Denisovan individual. *Sci. (New York, N.Y.)* **338**, 222–226, DOI: 10.1126/science.1224344 (2012).
2. Allentoft, M. E. *et al.* Population genomics of Bronze Age Eurasia. *Nature* **522**, 167–172, DOI: 10.1038/nature14507 (2015).
3. Haber, M. *et al.* Continuity and Admixture in the Last Five Millennia of Levantine History from Ancient Canaanite and Present-Day Lebanese Genome Sequences. *Am. J. Hum. Genet.* **101**, 274–282, DOI: 10.1016/j.ajhg.2017.06.013 (2017).
4. Haber, M. *et al.* A Transient Pulse of Genetic Admixture from the Crusaders in the Near East Identified from Ancient Genome Sequences. *Am. J. Hum. Genet.* **104**, 977–984, DOI: 10.1016/j.ajhg.2019.03.015 (2019).
5. Bergström, A. *et al.* Insights into human genetic variation and population history from 929 diverse genomes. *Sci. (New York, N.Y.)* **367**, eaay5012, DOI: 10.1126/science.aay5012 (2020).
6. Feldman, M. *et al.* Ancient DNA sheds light on the genetic origins of early Iron Age Philistines. *Sci. Adv.* **5**, eaax0061, DOI: 10.1126/sciadv.aax0061 (2019).
7. Lazaridis, I. *et al.* Ancient human genomes suggest three ancestral populations for present-day europeans. *Nature* **513**, 409–413 (2014).
8. Lazaridis, I. *et al.* Genetic origins of the Minoans and Mycenaeans. *Nature* **548**, 214–218, DOI: 10.1038/nature23310 (2017).
9. Agranat-Tamir, L. *et al.* The Genomic History of the Bronze Age Southern Levant. *Cell* **181**, 1146–1157.e11, DOI: 10.1016/j.cell.2020.04.024 (2020).
10. Narasimhan, V. M. *et al.* The formation of human populations in South and Central Asia. *Sci. (New York, N.Y.)* **365**, eaat7487, DOI: 10.1126/science.aat7487 (2019).
11. Skourtanioti, E. *et al.* Genomic History of Neolithic to Bronze Age Anatolia, Northern Levant, and Southern Caucasus. *Cell* **181**, 1158–1175.e28, DOI: 10.1016/j.cell.2020.04.044 (2020).
12. van den Brink, E. C. M. *et al.* A Late Bronze Age II clay coffin from Tel Shaddud in the Central Jezreel Valley, Israel: context and historical implications. *Levant* **49**, 105–135, DOI: 10.1080/00758914.2017.1368204 (2017). Publisher: Routledge _eprint: https://doi.org/10.1080/00758914.2017.1368204.
13. Damgaard, P. d. B. *et al.* 137 ancient human genomes from across the Eurasian steppes. *Nature* **557**, 369–374, DOI: 10.1038/s41586-018-0094-2 (2018).
14. de Barros Damgaard, P. *et al.* The first horse herders and the impact of early Bronze Age steppe expansions into Asia. *Sci. (New York, N.Y.)* **360**, eaar7711, DOI: 10.1126/science.aar7711 (2018).
15. Broushaki, F. *et al.* Early Neolithic genomes from the eastern Fertile Crescent. *Sci. (New York, N.Y.)* **353**, 499–503, DOI: 10.1126/science.aaf7943 (2016).
16. Reitsema, L. J. *et al.* The diverse genetic origins of a Classical period Greek army. *Proc. Natl. Acad. Sci. United States Am.* **119**, e2205272119, DOI: 10.1073/pnas.2205272119 (2022).
17. Paschou, P. *et al.* Maritime route of colonization of Europe. *Proc. Natl. Acad. Sci.* **111**, 9211–9216, DOI: 10.1073/pnas.1320811111 (2014). Publisher: Proceedings of the National Academy of Sciences.
