# Supplementary material for: Anatolian genetic ancestry in North Lebanese populations
Source: Sci Rep. 2024 Jul 5;14:15518. doi: 10.1038/s41598-024-66191-x (PMC11226446; doi:10.1038/s41598-024-66191-x)
Supplement: Supplementary file 2 — Supplementary Table S1. [file 41598_2024_66191_MOESM2_ESM.pdf]

Sample	Population	Reference	Years before 1950 CE
I7179	Ancient_Israel	AgranatTamirCell2020	3725
I7180	Ancient_Israel	AgranatTamirCell2020	3725
I7182	Ancient_Israel	AgranatTamirCell2020	3725
I7184	Ancient_Israel	AgranatTamirCell2020	3725
A_Mbuti-5.DG	Mbuti	MeyerScience2012	0
F38.SG	Ancient_Iran	BroushakiScience2016	2833
I0070	Ancient_Greece	LazaridisNature2017	4000
I0071	Ancient_Greece	LazaridisNature2017	4000
I0073	Ancient_Greece	LazaridisNature2017	4000
I0074	Ancient_Greece	LazaridisNature2017	4000
I9010	Ancient_Greece	LazaridisNature2017	3250
I9005	Ancient_Greece	LazaridisNature2017	4000
I9006	Ancient_Greece	LazaridisNature2017	3283
I9033	Ancient_Greece	LazaridisNature2017	3296
I9041	Ancient_Greece	LazaridisNature2017	3250
I9123	Ancient_Greece	LazaridisNature2017	3305
I9127	Ancient_Greece	LazaridisNature2017	3895
I9128	Ancient_Greece	LazaridisNature2017	3895
I9129	Ancient_Greece	LazaridisNature2017	3895
I9130	Ancient_Greece	LazaridisNature2017	3895
I9131	Ancient_Greece	LazaridisNature2017	3895
I2189_published	Ancient_Israel	AgranatTamirCell2020	3500
I2190	Ancient_Israel	AgranatTamirCell2020	3351
I2195	Ancient_Israel	AgranatTamirCell2020	3372
I2198	Ancient_Israel	AgranatTamirCell2020	3420
I2200	Ancient_Israel	AgranatTamirCell2020	3500
I2201	Ancient_Israel	AgranatTamirCell2020	2889
I1656	Ancient_Armenia	LazaridisNature2016	3397
RISE396.SG	Ancient_Armenia	AllentoftNature2015	3009
RISE397.SG	Ancient_Armenia	AllentoftNature2015	2908
RISE407.SG	Ancient_Armenia	AllentoftNature2015	2935
RISE408.SG	Ancient_Armenia	AllentoftNature2015	3050
RISE412.SG	Ancient_Armenia	AllentoftNature2015	3018
RISE413.SG	Ancient_Armenia	AllentoftNature2015	3764
RISE416.SG	Ancient_Armenia	AllentoftNature2015	3477
RISE423.SG	Ancient_Armenia	AllentoftNature2015	3251
I1934_published	Ancient_Israel	VanDenBrink2017	3200
I4517	Ancient_Israel	AgranatTamirCell2020	2951
I4518	Ancient_Israel	AgranatTamirCell2020	3375
I4519	Ancient_Israel	AgranatTamirCell2020	3425
I4525	Ancient_Israel	AgranatTamirCell2020	3500
I6463	Jordan	AgranatTamirCell2020	3300
I6565	Jordan	AgranatTamirCell2020	3300
I6461	Jordan	AgranatTamirCell2020	3300
I6462	Jordan	AgranatTamirCell2020	3300
I6464	Jordan	AgranatTamirCell2020	3300
I6564	Jordan	AgranatTamirCell2020	3300

I6566	Jordan	AgranatTamirCell2020	3300
I6567	Jordan	AgranatTamirCell2020	3300
I6569	Jordan	AgranatTamirCell2020	3300
I6570	Jordan	AgranatTamirCell2020	3300
I6572	Jordan	AgranatTamirCell2020	3300
I6460	Jordan	AgranatTamirCell2020	3300
I3986	Jordan	AgranatTamirCell2020	3300
I2327	Ancient_Iran	NarasimhanPattersonScie	3050
I3832	Ancient_Israel	AgranatTamirCell2020	3300
I3703	Jordan	AgranatTamirCell2020	3300
ERS1790729.SG	Ancient_Lebanon	HaberAJHG2017	3750
ERS1790731.SG	Ancient_Lebanon	HaberAJHG2017	3750
I6932	Ancient_Israel	AgranatTamirCell2020	3725
I6925	Ancient_Israel	AgranatTamirCell2020	3725
I6924	Ancient_Israel	AgranatTamirCell2020	3725
I6928	Ancient_Israel	AgranatTamirCell2020	3725
I7002	Ancient_Israel	AgranatTamirCell2020	3725
I6922	Ancient_Israel	AgranatTamirCell2020	3725
I6923	Ancient_Israel	AgranatTamirCell2020	3725
I7003	Ancient_Israel	AgranatTamirCell2020	3725
I10092	Ancient_Israel	AgranatTamirCell2020	3750
I10093	Ancient_Israel	AgranatTamirCell2020	3750
I10096	Ancient_Israel	AgranatTamirCell2020	3500
I10097	Ancient_Israel	AgranatTamirCell2020	3500
I10099	Ancient_Israel	AgranatTamirCell2020	3500
I10100	Ancient_Israel	AgranatTamirCell2020	3548
I10101	Ancient_Israel	AgranatTamirCell2020	3500
I10104	Ancient_Israel	AgranatTamirCell2020	3825
I10106	Ancient_Israel	AgranatTamirCell2020	3550
I10264	Ancient_Israel	AgranatTamirCell2020	3750
I10265	Ancient_Israel	AgranatTamirCell2020	3825
I10266	Ancient_Israel	AgranatTamirCell2020	3462
I10267	Ancient_Israel	AgranatTamirCell2020	3527
I10268	Ancient_Israel	AgranatTamirCell2020	3853
I10269	Ancient_Israel	AgranatTamirCell2020	3600
I10361	Ancient_Israel	AgranatTamirCell2020	3500
I7177	Ancient_Israel	AgranatTamirCell2020	3725
I8187	Ancient_Israel	AgranatTamirCell2020	3675
I8188	Ancient_Israel	AgranatTamirCell2020	3675
DA31.SG	Ancient_Armenia	DamgaardNature2018	3200
MA2197_final.SG	Ancient_Anatolia	DamgaardScience2018	2900
MA2198_final.SG	Ancient_Anatolia	DamgaardScience2018	2900
MA2200_final.SG	Ancient_Anatolia	DamgaardScience2018	3800
MA2203_final.SG	Ancient_Anatolia	DamgaardScience2018	3800
MA2205_final.SG	Ancient_Anatolia	DamgaardScience2018	3800
MA2206_final.SG	Ancient_Anatolia	DamgaardScience2018	3800
MA2208_final.SG	Ancient_Anatolia	DamgaardScience2018	3800
HGDP00471.SDG	Mbuti	BergstromScience2020	0

HGDP00462.SDG	Mbuti	BergstromScience2020	0
HGDP00450.SDG	Mbuti	BergstromScience2020	0
HGDP00463.SDG	Mbuti	BergstromScience2020	0
HGDP00467.SDG	Mbuti	BergstromScience2020	0
HGDP00478.SDG	Mbuti	BergstromScience2020	0
HGDP01081.SDG	Mbuti	BergstromScience2020	0
HGDP00984.SDG	Mbuti	BergstromScience2020	0
HGDP00449.SDG	Mbuti	BergstromScience2020	0
HGDP00476.SDG	Mbuti	BergstromScience2020	0
HGDP00474.SDG	Mbuti	BergstromScience2020	0
HGDP00982.SDG	Mbuti	BergstromScience2020	0
I10771	Ancient_Israel	AgranatTamirCell2020	3550
I10768	Ancient_Israel	AgranatTamirCell2020	3500
QED-12.SG	Ancient_Lebanon	HaberAJHG2018	1525
ASH008	Ancient_Israel	FeldmanScienceAdvances	3095
ASH029	Ancient_Israel	FeldmanScienceAdvances	3513
ASH033	Ancient_Israel	FeldmanScienceAdvances	3639
ASH034	Ancient_Israel	FeldmanScienceAdvances	3550
ASH066	Ancient_Israel	FeldmanScienceAdvances	3184
ASH067	Ancient_Israel	FeldmanScienceAdvances	3206
ASH068	Ancient_Israel	FeldmanScienceAdvances	3167
ASH087	Ancient_Israel	FeldmanScienceAdvances	3100
ASH135	Ancient_Israel	FeldmanScienceAdvances	3100
ASH2-3	Ancient_Israel	FeldmanScienceAdvances	3213
I10263_published	Ancient_Israel	AgranatTamirCell2020	3500
I10270_published	Ancient_Israel	AgranatTamirCell2020	3500
I10359_published	Ancient_Israel	AgranatTamirCell2020	3512
ALA009	Ancient_Anatolia	SkourtaniontiCell2020	3699
ALA015	Ancient_Anatolia	SkourtaniontiCell2020	3861
ALA016	Ancient_Anatolia	SkourtaniontiCell2020	3503
ALA017	Ancient_Anatolia	SkourtaniontiCell2020	3478
ALA023	Ancient_Anatolia	SkourtaniontiCell2020	3785
ALA024	Ancient_Anatolia	SkourtaniontiCell2020	3889
ALA035	Ancient_Anatolia	SkourtaniontiCell2020	3820
ALA037	Ancient_Anatolia	SkourtaniontiCell2020	3754
ALA038	Ancient_Anatolia	SkourtaniontiCell2020	3473
ALA084	Ancient_Anatolia	SkourtaniontiCell2020	3844
ETM001	Ancient_Syria	SkourtaniontiCell2020	3800
ETM003	Ancient_Syria	SkourtaniontiCell2020	2818
ETM004	Ancient_Syria	SkourtaniontiCell2020	3850
ETM005	Ancient_Syria	SkourtaniontiCell2020	3850
ETM006	Ancient_Syria	SkourtaniontiCell2020	3850
ETM014	Ancient_Syria	SkourtaniontiCell2020	3850
ETM015	Ancient_Syria	SkourtaniontiCell2020	3850
ETM016	Ancient_Syria	SkourtaniontiCell2020	3914
ETM017	Ancient_Syria	SkourtaniontiCell2020	3850
ETM018	Ancient_Syria	SkourtaniontiCell2020	4004
ETM021	Ancient_Syria	SkourtaniontiCell2020	3800

ETM025	Ancient_Syria	SkourtaniontiCell2020	3800
ETM026	Ancient_Syria	SkourtaniontiCell2020	3850
SFI-11.SG	Ancient_Lebanon	HaberAJHG2020	1984
SFI-12.SG	Ancient_Lebanon	HaberAJHG2020	2123
SFI-34.SG	Ancient_Lebanon	HaberAJHG2020	2385
SFI-35.SG	Ancient_Lebanon	HaberAJHG2020	2385
SFI-36.SG	Ancient_Lebanon	HaberAJHG2020	2385
SFI-39.SG	Ancient_Lebanon	HaberAJHG2020	2385
SFI-42.SG	Ancient_Lebanon	HaberAJHG2020	2415
SFI-43.SG	Ancient_Lebanon	HaberAJHG2020	2487
SFI-44.SG	Ancient_Lebanon	HaberAJHG2020	2385
SFI-45.SG	Ancient_Lebanon	HaberAJHG2020	2385
SFI-47.SG	Ancient_Lebanon	HaberAJHG2020	2385
SFI-5.SG	Ancient_Lebanon	HaberAJHG2020	2159
SFI-50.SG	Ancient_Lebanon	HaberAJHG2020	2385
SFI-55.SG	Ancient_Lebanon	HaberAJHG2020	2720
SFI-56.SG	Ancient_Lebanon	HaberAJHG2020	2720
ALA001_d	Ancient_Anatolia	SkourtaniontiCell2020	3378
ALA014_d	Ancient_Anatolia	SkourtaniontiCell2020	3629
Adana23117.HO	LazaridisNature2014	Turkey/Adana	0
Adana23112.HO	LazaridisNature2014	Turkey/Adana	0
Adana23136.HO	LazaridisNature2014	Turkey/Adana	0
Adana23113.HO	LazaridisNature2014	Turkey/Adana	0
Adana23114.HO	LazaridisNature2014	Turkey/Adana	0
Adana23147.HO	LazaridisNature2014	Turkey/Adana	0
Adana23150.HO	LazaridisNature2014	Turkey/Adana	0
Adana23108.HO	LazaridisNature2014	Turkey/Adana	0
Adana23144.HO	LazaridisNature2014	Turkey/Adana	0
Adana23133.HO	LazaridisNature2014	Turkey/Adana	0
Aydin18636.HO	LazaridisNature2014	Turkey/Aydin	0
Aydin18112.HO	LazaridisNature2014	Turkey/Aydin	0
Aydin18587.HO	LazaridisNature2014	Turkey/Aydin	0
Aydin18948.HO	LazaridisNature2014	Turkey/Aydin	0
Aydin18414.HO	LazaridisNature2014	Turkey/Aydin	0
Aydin18784.HO	LazaridisNature2014	Turkey/Aydin	0
Aydin18483.HO	LazaridisNature2014	Turkey/Aydin	0
Aydin18419.HO	LazaridisNature2014	Turkey/Aydin	0
Aydin18873.HO	LazaridisNature2014	Turkey/Aydin	0
Aydin18596.HO	LazaridisNature2014	Turkey/Aydin	0
Balikesir16837.HO	LazaridisNature2014	Turkey/Balikesir	0
Balikesir16514.HO	LazaridisNature2014	Turkey/Balikesir	0
Balikesir17006.HO	LazaridisNature2014	Turkey/Balikesir	0
Balikesir16558.HO	LazaridisNature2014	Turkey/Balikesir	0
Balikesir16675.HO	LazaridisNature2014	Turkey/Balikesir	0
Balikesir16790.HO	LazaridisNature2014	Turkey/Balikesir	0
Turkish8BA62.HO	LazaridisNature2014	Turkey/Balikesir	0

Balikesir16653.HO	LazaridisNature2014	Turkey/Balikesir	0
Balikesir16563.HO	LazaridisNature2014	Turkey/Balikesir	0
Balikesir16577.HO	LazaridisNature2014	Turkey/Balikesir	0
Balikesir16887.HO	LazaridisNature2014	Turkey/Balikesir	0
Istanbul25081.HO	LazaridisNature2014	Turkey/Istanbul	0
Istanbul19708.HO	LazaridisNature2014	Turkey/Istanbul	0
Istanbul20010.HO	LazaridisNature2014	Turkey/Istanbul	0
Istanbul25098.HO	LazaridisNature2014	Turkey/Istanbul	0
Istanbul19185.HO	LazaridisNature2014	Turkey/Istanbul	0
Istanbul19810.HO	LazaridisNature2014	Turkey/Istanbul	0
Istanbul17778.HO	LazaridisNature2014	Turkey/Istanbul	0
Istanbul15781.HO	LazaridisNature2014	Turkey/Istanbul	0
Istanbul20040.HO	LazaridisNature2014	Turkey/Istanbul	0
Istanbul25095.HO	LazaridisNature2014	Turkey/Istanbul	0
Kayseri23271.HO	LazaridisNature2014	Turkey/Kayseri	0
Kayseri24392.HO	LazaridisNature2014	Turkey/Kayseri	0
Kayseri24402.HO	LazaridisNature2014	Turkey/Kayseri	0
Kayseri24276.HO	LazaridisNature2014	Turkey/Kayseri	0
S_Turkish-1.DG	MallickNature2016	Turkey/Kayseri	0
Kayseri23967.HO	LazaridisNature2014	Turkey/Kayseri	0
