# Supplementary material for: Anatolian genetic ancestry in North Lebanese populations
Source: Sci Rep. 2024 Jul 5;14:15518. doi: 10.1038/s41598-024-66191-x (PMC11226446; doi:10.1038/s41598-024-66191-x)
Supplement: Supplementary file 3 — Supplementary Table S2. [file 41598_2024_66191_MOESM3_ESM.pdf]

Sample	Population	Platform
10AF233	Lebanon	Infinium Omni Express-24 v1.2 Bead Chip
10AH252	Syria	Infinium Omni Express-24 v1.2 Bead Chip
10AJ100	Syria	Infinium Omni Express-24 v1.2 Bead Chip
10AJ182	Lebanon	Infinium Omni Express-24 v1.2 Bead Chip
10AJ184	Lebanon	Infinium Omni Express-24 v1.2 Bead Chip
10AL119	Palestine	Infinium Omni Express-24 v1.2 Bead Chip
10AL120	Palestine	Infinium Omni Express-24 v1.2 Bead Chip
10AL171	Palestine	Infinium Omni Express-24 v1.2 Bead Chip
10AL34	KSA	Infinium Omni Express-24 v1.2 Bead Chip
10AM149	Iran	Infinium Omni Express-24 v1.2 Bead Chip
10AM180	Iran	Infinium Omni Express-24 v1.2 Bead Chip
10AM187	Iran	Infinium Omni Express-24 v1.2 Bead Chip
10AM58	Jordan	Infinium Omni Express-24 v1.2 Bead Chip
10AM65	Jordan	Infinium Omni Express-24 v1.2 Bead Chip
10AM74	Jordan	Infinium Omni Express-24 v1.2 Bead Chip
10AQ100	Iran	Infinium Omni Express-24 v1.2 Bead Chip
10AQ102	Iran	Infinium Omni Express-24 v1.2 Bead Chip
10AQ113	Libya	Infinium Omni Express-24 v1.2 Bead Chip
10AQ132	Armenia	Infinium Omni Express-24 v1.2 Bead Chip
10AQ135	Armenia	Infinium Omni Express-24 v1.2 Bead Chip
10AQ136	Armenia	Infinium Omni Express-24 v1.2 Bead Chip
10AQ137	Armenia	Infinium Omni Express-24 v1.2 Bead Chip
10AQ154	Armenia	Infinium Omni Express-24 v1.2 Bead Chip
10AQ45	Kuwait	Infinium Omni Express-24 v1.2 Bead Chip
10AQ60	Iran	Infinium Omni Express-24 v1.2 Bead Chip
10AQ66	Iran	Infinium Omni Express-24 v1.2 Bead Chip
10AQ68	Bahrein	Infinium Omni Express-24 v1.2 Bead Chip
10AQ69	Iran	Infinium Omni Express-24 v1.2 Bead Chip
10AQ75	Bahrein	Infinium Omni Express-24 v1.2 Bead Chip
10AQ86	Iran	Infinium Omni Express-24 v1.2 Bead Chip
10AQ99	Iran	Infinium Omni Express-24 v1.2 Bead Chip
10AR15	Syria	Infinium Omni Express-24 v1.2 Bead Chip
10BA60	Turkey	Infinium Omni Express-24 v1.2 Bead Chip
10BA65	Iraq	Infinium Omni Express-24 v1.2 Bead Chip
10BA66	Iraq	Infinium Omni Express-24 v1.2 Bead Chip
10R4	Lebanon	Infinium Omni Express-24 v1.2 Bead Chip
10R5	Lebanon	Infinium Omni Express-24 v1.2 Bead Chip
11AH326	Syria	Infinium Omni Express-24 v1.2 Bead Chip
11AJ100	Syria	Infinium Omni Express-24 v1.2 Bead Chip
11AJ152	Syria	Infinium Omni Express-24 v1.2 Bead Chip
11AJ185	Lebanon	Infinium Omni Express-24 v1.2 Bead Chip
11AJ49	Lebanon	Infinium Omni Express-24 v1.2 Bead Chip
11AL119	Palestine	Infinium Omni Express-24 v1.2 Bead Chip
11AL121	Palestine	Infinium Omni Express-24 v1.2 Bead Chip
11AL23	Palestine	Infinium Omni Express-24 v1.2 Bead Chip
11AM140	Iran	Infinium Omni Express-24 v1.2 Bead Chip
11AM149	Iran	Infinium Omni Express-24 v1.2 Bead Chip
11AM160	Iran	Infinium Omni Express-24 v1.2 Bead Chip
11AM180	Iran	Infinium Omni Express-24 v1.2 Bead Chip

11AM187	Iran	Infinium Omni Express-24 v1.2 Bead Chip
11AM58	Jordan	Infinium Omni Express-24 v1.2 Bead Chip
11AQ102	Iran	Infinium Omni Express-24 v1.2 Bead Chip
11AQ103	Iran	Infinium Omni Express-24 v1.2 Bead Chip
11AQ113	Libya	Infinium Omni Express-24 v1.2 Bead Chip
11AQ132	Armenia	Infinium Omni Express-24 v1.2 Bead Chip
11AQ135	Armenia	Infinium Omni Express-24 v1.2 Bead Chip
11AQ136	Armenia	Infinium Omni Express-24 v1.2 Bead Chip
11AQ137	Armenia	Infinium Omni Express-24 v1.2 Bead Chip
11AQ154	Armenia	Infinium Omni Express-24 v1.2 Bead Chip
11AQ155	Syria	Infinium Omni Express-24 v1.2 Bead Chip
11AQ45	Kuwait	Infinium Omni Express-24 v1.2 Bead Chip
11AQ64	Iran	Infinium Omni Express-24 v1.2 Bead Chip
11AQ66	Iran	Infinium Omni Express-24 v1.2 Bead Chip
11AQ69	Iran	Infinium Omni Express-24 v1.2 Bead Chip
11AR126	Iraq	Infinium Omni Express-24 v1.2 Bead Chip
11AR16	Syria	Infinium Omni Express-24 v1.2 Bead Chip
11AR27	syria	Infinium Omni Express-24 v1.2 Bead Chip
11BA18	Algeria	Infinium Omni Express-24 v1.2 Bead Chip
14BA18	Turkey	Infinium Omni Express-24 v1.2 Bead Chip
11BA65	Iraq	Infinium Omni Express-24 v1.2 Bead Chip
11BA66	Iraq	Infinium Omni Express-24 v1.2 Bead Chip
12AH252	Syria	Infinium Omni Express-24 v1.2 Bead Chip
12AJ118	Syria	Infinium Omni Express-24 v1.2 Bead Chip
12AJ177	Lebanon	Infinium Omni Express-24 v1.2 Bead Chip
12AJ185	Lebanon	Infinium Omni Express-24 v1.2 Bead Chip
12AJ49	Iraq	Infinium Omni Express-24 v1.2 Bead Chip
12AJ57	Lebanon	Infinium Omni Express-24 v1.2 Bead Chip
12AL117	Palestine	Infinium Omni Express-24 v1.2 Bead Chip
12AL119	Palestine	Infinium Omni Express-24 v1.2 Bead Chip
12AL16	Syria	Infinium Omni Express-24 v1.2 Bead Chip
12AL171	Palestine	Infinium Omni Express-24 v1.2 Bead Chip
12AL23	Palestine	Infinium Omni Express-24 v1.2 Bead Chip
12AL24	Palestine	Infinium Omni Express-24 v1.2 Bead Chip
12AM122	Iran	Infinium Omni Express-24 v1.2 Bead Chip
12AM149	Iran	Infinium Omni Express-24 v1.2 Bead Chip
12AM58	Jordan	Infinium Omni Express-24 v1.2 Bead Chip
12AM63	Jordan	Infinium Omni Express-24 v1.2 Bead Chip
12AQ102	Iran	Infinium Omni Express-24 v1.2 Bead Chip
12AQ131	Armenia	Infinium Omni Express-24 v1.2 Bead Chip
12AQ135	Armenia	Infinium Omni Express-24 v1.2 Bead Chip
12AQ136	Armenia	Infinium Omni Express-24 v1.2 Bead Chip
12AQ53	Iran	Infinium Omni Express-24 v1.2 Bead Chip
12AQ54	Iran	Infinium Omni Express-24 v1.2 Bead Chip
12AQ60	Iran	Infinium Omni Express-24 v1.2 Bead Chip
12AQ61	Iran	Infinium Omni Express-24 v1.2 Bead Chip
12AQ64	Iran	Infinium Omni Express-24 v1.2 Bead Chip
12AQ66	Iran	Infinium Omni Express-24 v1.2 Bead Chip
12AQ69	Iran	Infinium Omni Express-24 v1.2 Bead Chip
12AQ99	Iran	Infinium Omni Express-24 v1.2 Bead Chip

12AR16	Syria	Infinium Omni Express-24 v1.2 Bead Chip
12BA58	Turkey	Infinium Omni Express-24 v1.2 Bead Chip
12BA60	Turkey	Infinium Omni Express-24 v1.2 Bead Chip
12BA65	Iraq	Infinium Omni Express-24 v1.2 Bead Chip
12BA66	Iraq	Infinium Omni Express-24 v1.2 Bead Chip
12R40	Iraq	Infinium Omni Express-24 v1.2 Bead Chip
13AH252	Syria	Infinium Omni Express-24 v1.2 Bead Chip
13AH326	Syria	Infinium Omni Express-24 v1.2 Bead Chip
13AJ183	KSA	Infinium Omni Express-24 v1.2 Bead Chip
13AJ55	Lebanon	Infinium Omni Express-24 v1.2 Bead Chip
13AL121	Palestine	Infinium Omni Express-24 v1.2 Bead Chip
13AL23	Palestine	Infinium Omni Express-24 v1.2 Bead Chip
13AM103	Iran	Infinium Omni Express-24 v1.2 Bead Chip
13AM122	Iran	Infinium Omni Express-24 v1.2 Bead Chip
13AM149	Iran	Infinium Omni Express-24 v1.2 Bead Chip
13AM164	Iran	Infinium Omni Express-24 v1.2 Bead Chip
13AM185	Iran	Infinium Omni Express-24 v1.2 Bead Chip
13AM58	Jordan	Infinium Omni Express-24 v1.2 Bead Chip
13AM74	Jordan	Infinium Omni Express-24 v1.2 Bead Chip
13AQ100	Iran	Infinium Omni Express-24 v1.2 Bead Chip
13AQ111	Iran	Infinium Omni Express-24 v1.2 Bead Chip
13AQ131	Armenia	Infinium Omni Express-24 v1.2 Bead Chip
13AQ132	Armenia	Infinium Omni Express-24 v1.2 Bead Chip
13AQ136	Armenia	Infinium Omni Express-24 v1.2 Bead Chip
13AQ69	Iran	Infinium Omni Express-24 v1.2 Bead Chip
13AR15	Syria	Infinium Omni Express-24 v1.2 Bead Chip
13AR22	Lebanon	Infinium Omni Express-24 v1.2 Bead Chip
13AR27	syria	Infinium Omni Express-24 v1.2 Bead Chip
13BA59	Turkey	Infinium Omni Express-24 v1.2 Bead Chip
13BA60	Turkey	Infinium Omni Express-24 v1.2 Bead Chip
13BA65	Iraq	Infinium Omni Express-24 v1.2 Bead Chip
13BA66	Iraq	Infinium Omni Express-24 v1.2 Bead Chip
13R40	Iraq	Infinium Omni Express-24 v1.2 Bead Chip
13R59	Iraq	Infinium Omni Express-24 v1.2 Bead Chip
14AJ137	Iraq	Infinium Omni Express-24 v1.2 Bead Chip
14AJ180	Lebanon	Infinium Omni Express-24 v1.2 Bead Chip
14AJ183	Lebanon	Infinium Omni Express-24 v1.2 Bead Chip
14AJ68	Iraq	Infinium Omni Express-24 v1.2 Bead Chip
14AL121	Palestine	Infinium Omni Express-24 v1.2 Bead Chip
14AL24	Palestine	Infinium Omni Express-24 v1.2 Bead Chip
14AM122	Iran	Infinium Omni Express-24 v1.2 Bead Chip
14AM140	Iran	Infinium Omni Express-24 v1.2 Bead Chip
14AM149	Iran	Infinium Omni Express-24 v1.2 Bead Chip
14AM164	Iran	Infinium Omni Express-24 v1.2 Bead Chip
14AM66	Jordan	Infinium Omni Express-24 v1.2 Bead Chip
14AQ100	Iran	Infinium Omni Express-24 v1.2 Bead Chip
14AQ111	Iran	Infinium Omni Express-24 v1.2 Bead Chip
14AQ116	Libya	Infinium Omni Express-24 v1.2 Bead Chip
14AQ131	Armenia	Infinium Omni Express-24 v1.2 Bead Chip
14AQ132	Armenia	Infinium Omni Express-24 v1.2 Bead Chip

14AQ135	Armenia	Infinium Omni Express-24 v1.2 Bead Chip
14AQ136	Armenia	Infinium Omni Express-24 v1.2 Bead Chip
14AQ154	Armenia	Infinium Omni Express-24 v1.2 Bead Chip
14AQ38	Kuwait	Infinium Omni Express-24 v1.2 Bead Chip
14AQ45	KSA	Infinium Omni Express-24 v1.2 Bead Chip
14AQ60	Iran	Infinium Omni Express-24 v1.2 Bead Chip
14AQ61	Iran	Infinium Omni Express-24 v1.2 Bead Chip
14AQ68	Bahrein	Infinium Omni Express-24 v1.2 Bead Chip
14AQ69	Iran	Infinium Omni Express-24 v1.2 Bead Chip
14AQ71	Bahrein	Infinium Omni Express-24 v1.2 Bead Chip
14AQ75	Bahrein	Infinium Omni Express-24 v1.2 Bead Chip
14AR22	Lebanon	Infinium Omni Express-24 v1.2 Bead Chip
14BA18	Algeria	Infinium Omni Express-24 v1.2 Bead Chip
14BA60	Turkey	Infinium Omni Express-24 v1.2 Bead Chip
14R4	Lebanon	Infinium Omni Express-24 v1.2 Bead Chip
14R7	Syria	Infinium Omni Express-24 v1.2 Bead Chip
15AJ100	Syria	Infinium Omni Express-24 v1.2 Bead Chip
15AJ119	Syria	Infinium Omni Express-24 v1.2 Bead Chip
15AJ182	Lebanon	Infinium Omni Express-24 v1.2 Bead Chip
15AJ55	Lebanon	Infinium Omni Express-24 v1.2 Bead Chip
15AJ68	Iraq	Infinium Omni Express-24 v1.2 Bead Chip
15AL117	Palestine	Infinium Omni Express-24 v1.2 Bead Chip
15AL121	Palestine	Infinium Omni Express-24 v1.2 Bead Chip
15AL171	Palestine	Infinium Omni Express-24 v1.2 Bead Chip
15AL23	Palestine	Infinium Omni Express-24 v1.2 Bead Chip
15AL24	Palestine	Infinium Omni Express-24 v1.2 Bead Chip
15AM149	Iran	Infinium Omni Express-24 v1.2 Bead Chip
15AM185	Iran	Infinium Omni Express-24 v1.2 Bead Chip
15AM64	Jordan	Infinium Omni Express-24 v1.2 Bead Chip
15AM65	Jordan	Infinium Omni Express-24 v1.2 Bead Chip
15AM66	Jordan	Infinium Omni Express-24 v1.2 Bead Chip
15AQ111	Iran	Infinium Omni Express-24 v1.2 Bead Chip
15AQ132	Armenia	Infinium Omni Express-24 v1.2 Bead Chip
15AQ154	Armenia	Infinium Omni Express-24 v1.2 Bead Chip
15AQ21	Iran	Infinium Omni Express-24 v1.2 Bead Chip
15AQ45	Kuwait	Infinium Omni Express-24 v1.2 Bead Chip
15AQ60	Iran	Infinium Omni Express-24 v1.2 Bead Chip
15AQ74	Iran	Infinium Omni Express-24 v1.2 Bead Chip
15AR16	Syria	Infinium Omni Express-24 v1.2 Bead Chip
15AR27	syria	Infinium Omni Express-24 v1.2 Bead Chip
15BA57	Turkey	Infinium Omni Express-24 v1.2 Bead Chip
15BA58	Turkey	Infinium Omni Express-24 v1.2 Bead Chip
15BA59	Turkey	Infinium Omni Express-24 v1.2 Bead Chip
15BA60	Turkey	Infinium Omni Express-24 v1.2 Bead Chip
15BA61	Turkey	Infinium Omni Express-24 v1.2 Bead Chip
15BA65	Iraq	Infinium Omni Express-24 v1.2 Bead Chip
15R7	Syria	Infinium Omni Express-24 v1.2 Bead Chip
16AH326	Syria	Infinium Omni Express-24 v1.2 Bead Chip
16AJ100	Syria	Infinium Omni Express-24 v1.2 Bead Chip
16AJ177	Syria	Infinium Omni Express-24 v1.2 Bead Chip

16AL24	Palestine	Infinium Omni Express-24 v1.2 Bead Chip
16AM111	Iran	Infinium Omni Express-24 v1.2 Bead Chip
16AM122	Iran	Infinium Omni Express-24 v1.2 Bead Chip
16AM140	Iran	Infinium Omni Express-24 v1.2 Bead Chip
16AM185	Iran	Infinium Omni Express-24 v1.2 Bead Chip
16AM66	Jordan	Infinium Omni Express-24 v1.2 Bead Chip
16AM68	Jordan	Infinium Omni Express-24 v1.2 Bead Chip
16AQ111	Iran	Infinium Omni Express-24 v1.2 Bead Chip
16AQ131	Armenia	Infinium Omni Express-24 v1.2 Bead Chip
16AQ135	Armenia	Infinium Omni Express-24 v1.2 Bead Chip
16AQ137	Armenia	Infinium Omni Express-24 v1.2 Bead Chip
16AQ65	Iran	Infinium Omni Express-24 v1.2 Bead Chip
16AQ66	Iran	Infinium Omni Express-24 v1.2 Bead Chip
16AQ68	Bahrein	Infinium Omni Express-24 v1.2 Bead Chip
16AR22	Syria	Infinium Omni Express-24 v1.2 Bead Chip
16BA57	Turkey	Infinium Omni Express-24 v1.2 Bead Chip
16BA65	Iraq	Infinium Omni Express-24 v1.2 Bead Chip
17AH326	Syria	Infinium Omni Express-24 v1.2 Bead Chip
17AJ119	Syria	Infinium Omni Express-24 v1.2 Bead Chip
17AJ181	Syria	Infinium Omni Express-24 v1.2 Bead Chip
17AJ55	Lebanon	Infinium Omni Express-24 v1.2 Bead Chip
17AL23	Palestine	Infinium Omni Express-24 v1.2 Bead Chip
17AL24	Palestine	Infinium Omni Express-24 v1.2 Bead Chip
17AQ137	Lebanon	Infinium Omni Express-24 v1.2 Bead Chip
17BA66	Iraq	Infinium Omni Express-24 v1.2 Bead Chip
18AJ152	Syria	Infinium Omni Express-24 v1.2 Bead Chip
18AJ177	Lebanon	Infinium Omni Express-24 v1.2 Bead Chip
18AJ68	Iraq	Infinium Omni Express-24 v1.2 Bead Chip
18AL24	Palestine	Infinium Omni Express-24 v1.2 Bead Chip
18AQ113	Libya	Infinium Omni Express-24 v1.2 Bead Chip
18AQ38	Kuwait	Infinium Omni Express-24 v1.2 Bead Chip
18AQ44	Kuwait	Infinium Omni Express-24 v1.2 Bead Chip
18AQ75	Bahrein	Infinium Omni Express-24 v1.2 Bead Chip
18AR15	Syria	Infinium Omni Express-24 v1.2 Bead Chip
18BA59	Turkey	Infinium Omni Express-24 v1.2 Bead Chip
19AL16	Syria	Infinium Omni Express-24 v1.2 Bead Chip
19AL23	Palestine	Infinium Omni Express-24 v1.2 Bead Chip
19AL24	Palestine	Infinium Omni Express-24 v1.2 Bead Chip
19AQ37	Kuwait	Infinium Omni Express-24 v1.2 Bead Chip
19AQ93	KSA	Infinium Omni Express-24 v1.2 Bead Chip
19AR15	Syria	Infinium Omni Express-24 v1.2 Bead Chip
19BA18	Algeria	Infinium Omni Express-24 v1.2 Bead Chip
19BA59	Turkey	Infinium Omni Express-24 v1.2 Bead Chip
19BA66	Iraq	Infinium Omni Express-24 v1.2 Bead Chip
1AJ1	Lebanon	Infinium Omni Express-24 v1.2 Bead Chip
1AJ152	Lebanon	Infinium Omni Express-24 v1.2 Bead Chip
1AJ177	KSA	Infinium Omni Express-24 v1.2 Bead Chip
1AJ49	Lebanon	Infinium Omni Express-24 v1.2 Bead Chip
1AJ50	Lebanon	Infinium Omni Express-24 v1.2 Bead Chip
1AJ67	Iraq	Infinium Omni Express-24 v1.2 Bead Chip

1AL167A	KSA	Infinium Omni Express-24 v1.2 Bead Chip
1AL23	Palestine	Infinium Omni Express-24 v1.2 Bead Chip
1AM157	Iran	Infinium Omni Express-24 v1.2 Bead Chip
1AM65	Jordan	Infinium Omni Express-24 v1.2 Bead Chip
1AM76	Jordan	Infinium Omni Express-24 v1.2 Bead Chip
1AQ112	Iran	Infinium Omni Express-24 v1.2 Bead Chip
1AQ115	Lebanon	Infinium Omni Express-24 v1.2 Bead Chip
1AQ116	Libya	Infinium Omni Express-24 v1.2 Bead Chip
1AQ122	Lebanon	Infinium Omni Express-24 v1.2 Bead Chip
1AQ127	Lebanon	Infinium Omni Express-24 v1.2 Bead Chip
1AQ135	Armenia	Infinium Omni Express-24 v1.2 Bead Chip
1AQ136	Armenia	Infinium Omni Express-24 v1.2 Bead Chip
1AQ155	Armenia	Infinium Omni Express-24 v1.2 Bead Chip
1AQ44	KSA	Infinium Omni Express-24 v1.2 Bead Chip
1AQ64	Iran	Infinium Omni Express-24 v1.2 Bead Chip
1AQ65	Iran	Infinium Omni Express-24 v1.2 Bead Chip
1AQ66	Iran	Infinium Omni Express-24 v1.2 Bead Chip
1AQ99	Iran	Infinium Omni Express-24 v1.2 Bead Chip
1AR18	Syria	Infinium Omni Express-24 v1.2 Bead Chip
1BA2	Iraq	Infinium Omni Express-24 v1.2 Bead Chip
1BA59	Turkey	Infinium Omni Express-24 v1.2 Bead Chip
1BA60	Turkey	Infinium Omni Express-24 v1.2 Bead Chip
1BA61	Turkey	Infinium Omni Express-24 v1.2 Bead Chip
1BA66	Iraq	Infinium Omni Express-24 v1.2 Bead Chip
1BAJ137	Iraq	Infinium Omni Express-24 v1.2 Bead Chip
1R4	Lebanon	Infinium Omni Express-24 v1.2 Bead Chip
1R5	Libya	Infinium Omni Express-24 v1.2 Bead Chip
20AJ184	KSA	Infinium Omni Express-24 v1.2 Bead Chip
20AJ57	Iraq	Infinium Omni Express-24 v1.2 Bead Chip
20AL24	Palestine	Infinium Omni Express-24 v1.2 Bead Chip
20AQ37	KSA	Infinium Omni Express-24 v1.2 Bead Chip
20AR15	Syria	Infinium Omni Express-24 v1.2 Bead Chip
21AR18	Syria	Infinium Omni Express-24 v1.2 Bead Chip
21BA58	Turkey	Infinium Omni Express-24 v1.2 Bead Chip
21BA59	Turkey	Infinium Omni Express-24 v1.2 Bead Chip
21BA61	Turkey	Infinium Omni Express-24 v1.2 Bead Chip
22AL24	Palestine	Infinium Omni Express-24 v1.2 Bead Chip
22AR18	Syria	Infinium Omni Express-24 v1.2 Bead Chip
22BA57	Turkey	Infinium Omni Express-24 v1.2 Bead Chip
22BA58	Turkey	Infinium Omni Express-24 v1.2 Bead Chip
22BA59	Turkey	Infinium Omni Express-24 v1.2 Bead Chip
22BA61	Turkey	Infinium Omni Express-24 v1.2 Bead Chip
22BA62	Turkey	Infinium Omni Express-24 v1.2 Bead Chip
23AR15	Syria	Infinium Omni Express-24 v1.2 Bead Chip
23AR27	syria	Infinium Omni Express-24 v1.2 Bead Chip
23BA18	Algeria	Infinium Omni Express-24 v1.2 Bead Chip
23BA59	Turkey	Infinium Omni Express-24 v1.2 Bead Chip
23BA60	Turkey	Infinium Omni Express-24 v1.2 Bead Chip
23BA61	Turkey	Infinium Omni Express-24 v1.2 Bead Chip
24AL42	Jordan	Infinium Omni Express-24 v1.2 Bead Chip

24AR15	Syria	Infinium Omni Express-24 v1.2 Bead Chip
24AR18	Syria	Infinium Omni Express-24 v1.2 Bead Chip
24BA57	Turkey	Infinium Omni Express-24 v1.2 Bead Chip
24BA61	Turkey	Infinium Omni Express-24 v1.2 Bead Chip
26AH252	Syria	Infinium Omni Express-24 v1.2 Bead Chip
26AR15	Syria	Infinium Omni Express-24 v1.2 Bead Chip
28AR15	Syria	Infinium Omni Express-24 v1.2 Bead Chip
29AH252	Syria	Infinium Omni Express-24 v1.2 Bead Chip
2AE360	Lebanon	Infinium Omni Express-24 v1.2 Bead Chip
2AJ177	KSA	Infinium Omni Express-24 v1.2 Bead Chip
2AJ67	Iraq	Infinium Omni Express-24 v1.2 Bead Chip
2AL119	Palestine	Infinium Omni Express-24 v1.2 Bead Chip
2AL133	KSA	Infinium Omni Express-24 v1.2 Bead Chip
2AM198	Iran	Infinium Omni Express-24 v1.2 Bead Chip
2AM59	Jordan	Infinium Omni Express-24 v1.2 Bead Chip
2AM63	Jordan	Infinium Omni Express-24 v1.2 Bead Chip
2AM66	Jordan	Infinium Omni Express-24 v1.2 Bead Chip
2AQ114	Libya	Infinium Omni Express-24 v1.2 Bead Chip
2AQ132	Armenia	Infinium Omni Express-24 v1.2 Bead Chip
2AQ135	Armenia	Infinium Omni Express-24 v1.2 Bead Chip
2AQ136	Armenia	Infinium Omni Express-24 v1.2 Bead Chip
2AQ137	Armenia	Infinium Omni Express-24 v1.2 Bead Chip
2AQ154	Armenia	Infinium Omni Express-24 v1.2 Bead Chip
2AQ155	Armenia	Infinium Omni Express-24 v1.2 Bead Chip
2AQ191	Iraq	Infinium Omni Express-24 v1.2 Bead Chip
2AQ26	Iran	Infinium Omni Express-24 v1.2 Bead Chip
2AQ44	KSA	Infinium Omni Express-24 v1.2 Bead Chip
2AQ54	Iran	Infinium Omni Express-24 v1.2 Bead Chip
2AQ65	Iran	Infinium Omni Express-24 v1.2 Bead Chip
2AQ66	Iran	Infinium Omni Express-24 v1.2 Bead Chip
2AQ99	Iran	Infinium Omni Express-24 v1.2 Bead Chip
2AR16	Syria	Infinium Omni Express-24 v1.2 Bead Chip
2BA59	Turkey	Infinium Omni Express-24 v1.2 Bead Chip
2BA66	Iraq	Infinium Omni Express-24 v1.2 Bead Chip
2R40	Iraq	Infinium Omni Express-24 v1.2 Bead Chip
2R59	Iraq	Infinium Omni Express-24 v1.2 Bead Chip
2R7	Lebanon	Infinium Omni Express-24 v1.2 Bead Chip
3AH326	Syria	Infinium Omni Express-24 v1.2 Bead Chip
3AJ112	Syria	Infinium Omni Express-24 v1.2 Bead Chip
3AJ116	Syria	Infinium Omni Express-24 v1.2 Bead Chip
3AJ118	Syria	Infinium Omni Express-24 v1.2 Bead Chip
3AJ120	Syria	Infinium Omni Express-24 v1.2 Bead Chip
3AJ177	KSA	Infinium Omni Express-24 v1.2 Bead Chip
3AJ185	Lebanon	Infinium Omni Express-24 v1.2 Bead Chip
3AJ67	Iraq	Infinium Omni Express-24 v1.2 Bead Chip
3AL119	Palestine	Infinium Omni Express-24 v1.2 Bead Chip
3AM58	Jordan	Infinium Omni Express-24 v1.2 Bead Chip
3AM63	Jordan	Infinium Omni Express-24 v1.2 Bead Chip
3AM68	Jordan	Infinium Omni Express-24 v1.2 Bead Chip
3AM77	Jordan	Infinium Omni Express-24 v1.2 Bead Chip

3AQ100	Iran	Infinium Omni Express-24 v1.2 Bead Chip
3AQ101	Iran	Infinium Omni Express-24 v1.2 Bead Chip
3AQ103	Iran	Infinium Omni Express-24 v1.2 Bead Chip
3AQ112	Iran	Infinium Omni Express-24 v1.2 Bead Chip
3AQ114	Libya	Infinium Omni Express-24 v1.2 Bead Chip
3AQ131	Armenia	Infinium Omni Express-24 v1.2 Bead Chip
3AQ135	Armenia	Infinium Omni Express-24 v1.2 Bead Chip
3AQ136	Armenia	Infinium Omni Express-24 v1.2 Bead Chip
3AQ137	Armenia	Infinium Omni Express-24 v1.2 Bead Chip
3AQ21	Iran	Infinium Omni Express-24 v1.2 Bead Chip
3AQ38	Kuwait	Infinium Omni Express-24 v1.2 Bead Chip
3AQ44	Kuwait	Infinium Omni Express-24 v1.2 Bead Chip
3AQ61	Iran	Infinium Omni Express-24 v1.2 Bead Chip
3AQ65	Iran	Infinium Omni Express-24 v1.2 Bead Chip
3AQ66	Iran	Infinium Omni Express-24 v1.2 Bead Chip
3AR16	Syria	Infinium Omni Express-24 v1.2 Bead Chip
3BA58	Turkey	Infinium Omni Express-24 v1.2 Bead Chip
3BA62	Turkey	Infinium Omni Express-24 v1.2 Bead Chip
3IBA2	KSA	Infinium Omni Express-24 v1.2 Bead Chip
3R5	Libya	Infinium Omni Express-24 v1.2 Bead Chip
4AJ112	Syria	Infinium Omni Express-24 v1.2 Bead Chip
4AJ120	Syria	Infinium Omni Express-24 v1.2 Bead Chip
4AJ152	Lebanon	Infinium Omni Express-24 v1.2 Bead Chip
4AJ177	KSA	Infinium Omni Express-24 v1.2 Bead Chip
4AJ178	Lebanon	Infinium Omni Express-24 v1.2 Bead Chip
4AJ183	Lebanon	Infinium Omni Express-24 v1.2 Bead Chip
4AL117	Palestine	Infinium Omni Express-24 v1.2 Bead Chip
4AL119	Palestine	Infinium Omni Express-24 v1.2 Bead Chip
4AL123	Palestine	Infinium Omni Express-24 v1.2 Bead Chip
4AL23	Palestine	Infinium Omni Express-24 v1.2 Bead Chip
4AL78	Iraq	Infinium Omni Express-24 v1.2 Bead Chip
4AM100	Iran	Infinium Omni Express-24 v1.2 Bead Chip
4AM140	Iran	Infinium Omni Express-24 v1.2 Bead Chip
4AM149	Iran	Infinium Omni Express-24 v1.2 Bead Chip
4AM157	Iran	Infinium Omni Express-24 v1.2 Bead Chip
4AM187	Iran	Infinium Omni Express-24 v1.2 Bead Chip
4AM64	Jordan	Infinium Omni Express-24 v1.2 Bead Chip
4AQ101	Iran	Infinium Omni Express-24 v1.2 Bead Chip
4AQ131	Armenia	Infinium Omni Express-24 v1.2 Bead Chip
4AQ135	Armenia	Infinium Omni Express-24 v1.2 Bead Chip
4AQ154	Armenia	Infinium Omni Express-24 v1.2 Bead Chip
4AQ155	Armenia	Infinium Omni Express-24 v1.2 Bead Chip
4AQ26	Iran	Infinium Omni Express-24 v1.2 Bead Chip
4AQ38	KSA	Infinium Omni Express-24 v1.2 Bead Chip
4AQ64	Iran	Infinium Omni Express-24 v1.2 Bead Chip
4AQ68	Bahrein	Infinium Omni Express-24 v1.2 Bead Chip
4AQ75	Bahrein	Infinium Omni Express-24 v1.2 Bead Chip
4AQ99	Iran	Infinium Omni Express-24 v1.2 Bead Chip
4BA59	Turkey	Infinium Omni Express-24 v1.2 Bead Chip
4BA66	Iraq	Infinium Omni Express-24 v1.2 Bead Chip

4R114	Iraq	Infinium Omni Express-24 v1.2 Bead Chip
4R5	Lebanon	Infinium Omni Express-24 v1.2 Bead Chip
4R7	Lebanon	Infinium Omni Express-24 v1.2 Bead Chip
5AH252	Syria	Infinium Omni Express-24 v1.2 Bead Chip
5AJ1	Iraq	Infinium Omni Express-24 v1.2 Bead Chip
5AJ100	Iraq	Infinium Omni Express-24 v1.2 Bead Chip
5AJ112	Syria	Infinium Omni Express-24 v1.2 Bead Chip
5AJ177	Lebanon	Infinium Omni Express-24 v1.2 Bead Chip
5AJ185	Lebanon	Infinium Omni Express-24 v1.2 Bead Chip
5AJ55	Lebanon	Infinium Omni Express-24 v1.2 Bead Chip
5AJ67	Iraq	Infinium Omni Express-24 v1.2 Bead Chip
5AL117	Palestine	Infinium Omni Express-24 v1.2 Bead Chip
5AL121	Palestine	Infinium Omni Express-24 v1.2 Bead Chip
5AL171	Palestine	Infinium Omni Express-24 v1.2 Bead Chip
5AL23	Palestine	Infinium Omni Express-24 v1.2 Bead Chip
5AL24	Palestine	Infinium Omni Express-24 v1.2 Bead Chip
5AM103	Iran	Infinium Omni Express-24 v1.2 Bead Chip
5AM111	Iran	Infinium Omni Express-24 v1.2 Bead Chip
5AM123	Iran	Infinium Omni Express-24 v1.2 Bead Chip
5AM140	Iran	Infinium Omni Express-24 v1.2 Bead Chip
5AM149	Iran	Infinium Omni Express-24 v1.2 Bead Chip
5AM187	Iran	Infinium Omni Express-24 v1.2 Bead Chip
5AM60	Jordan	Infinium Omni Express-24 v1.2 Bead Chip
5AM66	Jordan	Infinium Omni Express-24 v1.2 Bead Chip
5AM68	Jordan	Infinium Omni Express-24 v1.2 Bead Chip
5AQ103	Iran	Infinium Omni Express-24 v1.2 Bead Chip
5AQ12	Iran	Infinium Omni Express-24 v1.2 Bead Chip
5AQ135	Armenia	Infinium Omni Express-24 v1.2 Bead Chip
5AQ137	Armenia	Infinium Omni Express-24 v1.2 Bead Chip
5AQ154	Armenia	Infinium Omni Express-24 v1.2 Bead Chip
5AQ26	Iran	Infinium Omni Express-24 v1.2 Bead Chip
5AQ60	Iran	Infinium Omni Express-24 v1.2 Bead Chip
5AQ61	Iran	Infinium Omni Express-24 v1.2 Bead Chip
5AQ65	Iran	Infinium Omni Express-24 v1.2 Bead Chip
5AQ66	Iran	Infinium Omni Express-24 v1.2 Bead Chip
5AQ7	Iran	Infinium Omni Express-24 v1.2 Bead Chip
5AQ70	Iran	Infinium Omni Express-24 v1.2 Bead Chip
5AQ99	Iran	Infinium Omni Express-24 v1.2 Bead Chip
5AR16	Syria	Infinium Omni Express-24 v1.2 Bead Chip
5AS15	Syria	Infinium Omni Express-24 v1.2 Bead Chip
5B44	Syria	Infinium Omni Express-24 v1.2 Bead Chip
5BA66	Iraq	Infinium Omni Express-24 v1.2 Bead Chip
5R158	Lebanon	Infinium Omni Express-24 v1.2 Bead Chip
6AH252	Syria	Infinium Omni Express-24 v1.2 Bead Chip
6AJ118	Syria	Infinium Omni Express-24 v1.2 Bead Chip
6AJ181	Lebanon	Infinium Omni Express-24 v1.2 Bead Chip
6AJ55	Lebanon	Infinium Omni Express-24 v1.2 Bead Chip
6AL120	Palestine	Infinium Omni Express-24 v1.2 Bead Chip
6AL23	Palestine	Infinium Omni Express-24 v1.2 Bead Chip
6AM121	Iran	Infinium Omni Express-24 v1.2 Bead Chip

6AM140	Iran	Infinium Omni Express-24 v1.2 Bead Chip
6AM149	Iran	Infinium Omni Express-24 v1.2 Bead Chip
6AM187	Iran	Infinium Omni Express-24 v1.2 Bead Chip
6AM65	Jordan	Infinium Omni Express-24 v1.2 Bead Chip
6AM74	Jordan	Infinium Omni Express-24 v1.2 Bead Chip
6AQ113	Libya	Infinium Omni Express-24 v1.2 Bead Chip
6AQ131	Armenia	Infinium Omni Express-24 v1.2 Bead Chip
6AQ132	Armenia	Infinium Omni Express-24 v1.2 Bead Chip
6AQ154	Armenia	Infinium Omni Express-24 v1.2 Bead Chip
6AQ26	Iran	Infinium Omni Express-24 v1.2 Bead Chip
6AQ65	Iran	Infinium Omni Express-24 v1.2 Bead Chip
6AQ66	Iran	Infinium Omni Express-24 v1.2 Bead Chip
6AQ71	Bahrein	Infinium Omni Express-24 v1.2 Bead Chip
6AQ99	Iran	Infinium Omni Express-24 v1.2 Bead Chip
6AR15	Syria	Infinium Omni Express-24 v1.2 Bead Chip
6AR16	Syria	Infinium Omni Express-24 v1.2 Bead Chip
6AR20	Armenia	Infinium Omni Express-24 v1.2 Bead Chip
6AR27	syria	Infinium Omni Express-24 v1.2 Bead Chip
6BA1	Iraq	Infinium Omni Express-24 v1.2 Bead Chip
6BA58	Turkey	Infinium Omni Express-24 v1.2 Bead Chip
6BA66	Iraq	Infinium Omni Express-24 v1.2 Bead Chip
6R172	KSA	Infinium Omni Express-24 v1.2 Bead Chip
6R35	KSA	Infinium Omni Express-24 v1.2 Bead Chip
6R4	Lebanon	Infinium Omni Express-24 v1.2 Bead Chip
7AJ100	Iraq	Infinium Omni Express-24 v1.2 Bead Chip
7AJ127	KSA	Infinium Omni Express-24 v1.2 Bead Chip
7AJ152	Iraq	Infinium Omni Express-24 v1.2 Bead Chip
7AJ182	KSA	Infinium Omni Express-24 v1.2 Bead Chip
7AJ55	Iraq	Infinium Omni Express-24 v1.2 Bead Chip
7AL119	Palestine	Infinium Omni Express-24 v1.2 Bead Chip
7AL120	Palestine	Infinium Omni Express-24 v1.2 Bead Chip
7AL171	Palestine	Infinium Omni Express-24 v1.2 Bead Chip
7AL23	Palestine	Infinium Omni Express-24 v1.2 Bead Chip
7AL45	KSA	Infinium Omni Express-24 v1.2 Bead Chip
7AM140	Iran	Infinium Omni Express-24 v1.2 Bead Chip
7AM153	Iran	Infinium Omni Express-24 v1.2 Bead Chip
7AM164	Iran	Infinium Omni Express-24 v1.2 Bead Chip
7AM187	Iran	Infinium Omni Express-24 v1.2 Bead Chip
7AM66	Jordan	Infinium Omni Express-24 v1.2 Bead Chip
7AM94	Iran	Infinium Omni Express-24 v1.2 Bead Chip
7AQ101	Iran	Infinium Omni Express-24 v1.2 Bead Chip
7AQ115	Lebanon	Infinium Omni Express-24 v1.2 Bead Chip
7AQ117	Libya	Infinium Omni Express-24 v1.2 Bead Chip
7AQ136	Armenia	Infinium Omni Express-24 v1.2 Bead Chip
7AQ137	Armenia	Infinium Omni Express-24 v1.2 Bead Chip
7AQ154	Armenia	Infinium Omni Express-24 v1.2 Bead Chip
7AQ60	Iran	Infinium Omni Express-24 v1.2 Bead Chip
7AQ65	Iran	Infinium Omni Express-24 v1.2 Bead Chip
7AQ66	Iran	Infinium Omni Express-24 v1.2 Bead Chip
7AQ69	Iran	Infinium Omni Express-24 v1.2 Bead Chip

7AR15	Syria	Infinium Omni Express-24 v1.2 Bead Chip
7BA59	Turkey	Infinium Omni Express-24 v1.2 Bead Chip
7BA65	Iraq	Infinium Omni Express-24 v1.2 Bead Chip
7BA66	Iraq	Infinium Omni Express-24 v1.2 Bead Chip
7R4	Lebanon	Infinium Omni Express-24 v1.2 Bead Chip
7R7	Lebanon	Infinium Omni Express-24 v1.2 Bead Chip
8AH252	Syria	Infinium Omni Express-24 v1.2 Bead Chip
8AJ184	Lebanon	Infinium Omni Express-24 v1.2 Bead Chip
8AJ57	Lebanon	Infinium Omni Express-24 v1.2 Bead Chip
8AL120	Palestine	Infinium Omni Express-24 v1.2 Bead Chip
8AL171	Palestine	Infinium Omni Express-24 v1.2 Bead Chip
8AM100	Iran	Infinium Omni Express-24 v1.2 Bead Chip
8AM149	Iran	Infinium Omni Express-24 v1.2 Bead Chip
8AM164	Iran	Infinium Omni Express-24 v1.2 Bead Chip
8AM187	Iran	Infinium Omni Express-24 v1.2 Bead Chip
8AM198	Iran	Infinium Omni Express-24 v1.2 Bead Chip
8AM45	Jordan	Infinium Omni Express-24 v1.2 Bead Chip
8AM66	Jordan	Infinium Omni Express-24 v1.2 Bead Chip
8AM74	Jordan	Infinium Omni Express-24 v1.2 Bead Chip
8AM77	Jordan	Infinium Omni Express-24 v1.2 Bead Chip
8AQ101	Iran	Infinium Omni Express-24 v1.2 Bead Chip
8AQ113	Libya	Infinium Omni Express-24 v1.2 Bead Chip
8AQ132	Armenia	Infinium Omni Express-24 v1.2 Bead Chip
8AQ136	Armenia	Infinium Omni Express-24 v1.2 Bead Chip
8AQ154	Armenia	Infinium Omni Express-24 v1.2 Bead Chip
8AQ45	Kuwait	Infinium Omni Express-24 v1.2 Bead Chip
8AQ64	Iran	Infinium Omni Express-24 v1.2 Bead Chip
8AQ65	Iran	Infinium Omni Express-24 v1.2 Bead Chip
8AQ66	Iran	Infinium Omni Express-24 v1.2 Bead Chip
8AQ75	Bahrein	Infinium Omni Express-24 v1.2 Bead Chip
8AQ99	Iran	Infinium Omni Express-24 v1.2 Bead Chip
8AR15	Syria	Infinium Omni Express-24 v1.2 Bead Chip
8BA62	Turkey	Infinium Omni Express-24 v1.2 Bead Chip
8BA66	Iraq	Infinium Omni Express-24 v1.2 Bead Chip
9AF233	Lebanon	Infinium Omni Express-24 v1.2 Bead Chip
9AL119	Palestine	Infinium Omni Express-24 v1.2 Bead Chip
9AL120	Palestine	Infinium Omni Express-24 v1.2 Bead Chip
9AL171	Palestine	Infinium Omni Express-24 v1.2 Bead Chip
9AM100	Iran	Infinium Omni Express-24 v1.2 Bead Chip
9AM140	Iran	Infinium Omni Express-24 v1.2 Bead Chip
9AM141	Iran	Infinium Omni Express-24 v1.2 Bead Chip
9AM149	Iran	Infinium Omni Express-24 v1.2 Bead Chip
9AM164	Iran	Infinium Omni Express-24 v1.2 Bead Chip
9AM185	Iran	Infinium Omni Express-24 v1.2 Bead Chip
9AM187	Iran	Infinium Omni Express-24 v1.2 Bead Chip
9AM58	Jordan	Infinium Omni Express-24 v1.2 Bead Chip
9AM68	Jordan	Infinium Omni Express-24 v1.2 Bead Chip
9AQ100	Iran	Infinium Omni Express-24 v1.2 Bead Chip
9AQ101	Iran	Infinium Omni Express-24 v1.2 Bead Chip
9AQ102	Iran	Infinium Omni Express-24 v1.2 Bead Chip

9AQ131	Armenia	Infinium Omni Express-24 v1.2 Bead Chip
9AQ135	Armenia	Infinium Omni Express-24 v1.2 Bead Chip
9AQ136	Armenia	Infinium Omni Express-24 v1.2 Bead Chip
9AQ137	Armenia	Infinium Omni Express-24 v1.2 Bead Chip
9AQ154	Armenia	Infinium Omni Express-24 v1.2 Bead Chip
9AQ61	Iran	Infinium Omni Express-24 v1.2 Bead Chip
9AQ64	Iran	Infinium Omni Express-24 v1.2 Bead Chip
9AQ69	Iran	Infinium Omni Express-24 v1.2 Bead Chip
9AQ86	Iran	Infinium Omni Express-24 v1.2 Bead Chip
9AR15	Syria	Infinium Omni Express-24 v1.2 Bead Chip
9AR16	Syria	Infinium Omni Express-24 v1.2 Bead Chip
9BA58	Turkey	Infinium Omni Express-24 v1.2 Bead Chip
9BA65	Iraq	Infinium Omni Express-24 v1.2 Bead Chip
9BA66	Iraq	Infinium Omni Express-24 v1.2 Bead Chip
9R180	Iraq	Infinium Omni Express-24 v1.2 Bead Chip
AMR7	Yemen	Infinium Omni Express-24 v1.2 Bead Chip
CYP15	Cyprus	Infinium Omni Express-24 v1.2 Bead Chip
CYP19	Cyprus	Infinium Omni Express-24 v1.2 Bead Chip
CYP20	Cyprus	Infinium Omni Express-24 v1.2 Bead Chip
CYP22	Cyprus	Infinium Omni Express-24 v1.2 Bead Chip
CYP25	Cyprus	Infinium Omni Express-24 v1.2 Bead Chip
CYP27	Cyprus	Infinium Omni Express-24 v1.2 Bead Chip
CYP30	Cyprus	Infinium Omni Express-24 v1.2 Bead Chip
CYP32	Cyprus	Infinium Omni Express-24 v1.2 Bead Chip
CYP34	Cyprus	Infinium Omni Express-24 v1.2 Bead Chip
CYP35	Cyprus	Infinium Omni Express-24 v1.2 Bead Chip
CYP37	Cyprus	Infinium Omni Express-24 v1.2 Bead Chip
CYP39	Cyprus	Infinium Omni Express-24 v1.2 Bead Chip
CYP41	Cyprus	Infinium Omni Express-24 v1.2 Bead Chip
CYP42	Cyprus	Infinium Omni Express-24 v1.2 Bead Chip
CYP45	Cyprus	Infinium Omni Express-24 v1.2 Bead Chip
CYP46	Cyprus	Infinium Omni Express-24 v1.2 Bead Chip
HAJ6	Yemen	Infinium Omni Express-24 v1.2 Bead Chip
HAJ7	Yemen	Infinium Omni Express-24 v1.2 Bead Chip
HDR20	Yemen	Infinium Omni Express-24 v1.2 Bead Chip
HDR22	Yemen	Infinium Omni Express-24 v1.2 Bead Chip
IBB4	Yemen	Infinium Omni Express-24 v1.2 Bead Chip
MHW2	Yemen	Infinium Omni Express-24 v1.2 Bead Chip
MRB8	Yemen	Infinium Omni Express-24 v1.2 Bead Chip
RSA10	Yemen	Infinium Omni Express-24 v1.2 Bead Chip
RSA13	Yemen	Infinium Omni Express-24 v1.2 Bead Chip
RSA14	Yemen	Infinium Omni Express-24 v1.2 Bead Chip
RSA16	Yemen	Infinium Omni Express-24 v1.2 Bead Chip
RSA24	Yemen	Infinium Omni Express-24 v1.2 Bead Chip
RSA3	Yemen	Infinium Omni Express-24 v1.2 Bead Chip
RSA4	Yemen	Infinium Omni Express-24 v1.2 Bead Chip
SHB10	Yemen	Infinium Omni Express-24 v1.2 Bead Chip
TIZ1	Yemen	Infinium Omni Express-24 v1.2 Bead Chip
TIZ17	Yemen	Infinium Omni Express-24 v1.2 Bead Chip
TIZ2	Yemen	Infinium Omni Express-24 v1.2 Bead Chip

Al001	Algeria	Human 1M-duo v3.0 array
Al002	Algeria	Human 1M-duo v3.0 array
Al003	Algeria	Human 1M-duo v3.0 array
Eg001	Egypt	Human 1M-duo v3.0 array
Eg002	Egypt	Human 1M-duo v3.0 array
Eg003	Egypt	Human 1M-duo v3.0 array
Eg004	Egypt	Human 1M-duo v3.0 array
Eg005	Egypt	Human 1M-duo v3.0 array
Eg006	Egypt	Human 1M-duo v3.0 array
Eg007	Egypt	Human 1M-duo v3.0 array
Eg008	Egypt	Human 1M-duo v3.0 array
Eg009	Egypt	Human 1M-duo v3.0 array
Eg010	Egypt	Human 1M-duo v3.0 array
Eg011	Egypt	Human 1M-duo v3.0 array
Eg012	Egypt	Human 1M-duo v3.0 array
Eg013	Egypt	Human 1M-duo v3.0 array
Eg014	Egypt	Human 1M-duo v3.0 array
Eg015	Egypt	Human 1M-duo v3.0 array
Eg016	Egypt	Human 1M-duo v3.0 array
Eg017	Egypt	Human 1M-duo v3.0 array
Eg018	Egypt	Human 1M-duo v3.0 array
Eg019	Egypt	Human 1M-duo v3.0 array
Eg020	Egypt	Human 1M-duo v3.0 array
Eg021	Egypt	Human 1M-duo v3.0 array
Eg022	Egypt	Human 1M-duo v3.0 array
Eg023	Egypt	Human 1M-duo v3.0 array
Eg024	Egypt	Human 1M-duo v3.0 array
Eg025	Egypt	Human 1M-duo v3.0 array
Eg026	Egypt	Human 1M-duo v3.0 array
Eg027	Egypt	Human 1M-duo v3.0 array
Eg028	Egypt	Human 1M-duo v3.0 array
Eg029	Egypt	Human 1M-duo v3.0 array
Eg030	Egypt	Human 1M-duo v3.0 array
Eg031	Egypt	Human 1M-duo v3.0 array
Eg032	Egypt	Human 1M-duo v3.0 array
Eg033	Egypt	Human 1M-duo v3.0 array
Gr001	Greece	Human 1M-duo v3.0 array
Gr002	Greece	Human 1M-duo v3.0 array
Gr003	Greece	Human 1M-duo v3.0 array
Jo001	Jordan	Human 1M-duo v3.0 array
Jo002	Jordan	Human 1M-duo v3.0 array
Jo003	Jordan	Human 1M-duo v3.0 array
Jo004	Jordan	Human 1M-duo v3.0 array
Jo005	Jordan	Human 1M-duo v3.0 array
Jo006	Jordan	Human 1M-duo v3.0 array
KSA1	KSA	Human 1M-duo v3.0 array
KSA2	KSA	Human 1M-duo v3.0 array
KSA3	KSA	Human 1M-duo v3.0 array
KSA4	KSA	Human 1M-duo v3.0 array
KSA5	KSA	Human 1M-duo v3.0 array

KSA6	KSA	Human 1M-duo v3.0 array
KSA7	KSA	Human 1M-duo v3.0 array
Lb001	Lebanon	Human 1M-duo v3.0 array
Lb002	Lebanon	Human 1M-duo v3.0 array
Lb003	Lebanon	Human 1M-duo v3.0 array
Lb004	Lebanon	Human 1M-duo v3.0 array
Lb005	Lebanon	Human 1M-duo v3.0 array
Lb006	Lebanon	Human 1M-duo v3.0 array
Lb007	Lebanon	Human 1M-duo v3.0 array
Lb008	Lebanon	Human 1M-duo v3.0 array
Mo001	Morocco	Human 1M-duo v3.0 array
Om001	Oman	Human 1M-duo v3.0 array
Om002	Oman	Human 1M-duo v3.0 array
Om003	Oman	Human 1M-duo v3.0 array
Om004	Oman	Human 1M-duo v3.0 array
Om005	Oman	Human 1M-duo v3.0 array
Om006	Oman	Human 1M-duo v3.0 array
Om007	Oman	Human 1M-duo v3.0 array
Om008	Oman	Human 1M-duo v3.0 array
Om009	Oman	Human 1M-duo v3.0 array
Pal001	Palestine	Human 1M-duo v3.0 array
Pal002	Palestine	Human 1M-duo v3.0 array
Pal003	Palestine	Human 1M-duo v3.0 array
Pal004	Palestine	Human 1M-duo v3.0 array
Pal005	Palestine	Human 1M-duo v3.0 array
Pal006	Palestine	Human 1M-duo v3.0 array
Pal007	Palestine	Human 1M-duo v3.0 array
Pal008	Palestine	Human 1M-duo v3.0 array
Pal009	Palestine	Human 1M-duo v3.0 array
Pal010	Palestine	Human 1M-duo v3.0 array
Pal011	Palestine	Human 1M-duo v3.0 array
Pal012	Palestine	Human 1M-duo v3.0 array
Pal013	Palestine	Human 1M-duo v3.0 array
Pal014	Palestine	Human 1M-duo v3.0 array
Pal015	Palestine	Human 1M-duo v3.0 array
Pal016	Palestine	Human 1M-duo v3.0 array
Pal017	Palestine	Human 1M-duo v3.0 array
Pal018	Palestine	Human 1M-duo v3.0 array
Pal019	Palestine	Human 1M-duo v3.0 array
Pal020	Palestine	Human 1M-duo v3.0 array
Pal021	Palestine	Human 1M-duo v3.0 array
Pal022	Palestine	Human 1M-duo v3.0 array
Pal023	Palestine	Human 1M-duo v3.0 array
Pal024	Palestine	Human 1M-duo v3.0 array
Pal025	Palestine	Human 1M-duo v3.0 array
Pal026	Palestine	Human 1M-duo v3.0 array
Pal027	Palestine	Human 1M-duo v3.0 array
Pal028	Palestine	Human 1M-duo v3.0 array
Pal029	Palestine	Human 1M-duo v3.0 array
Pal030	Palestine	Human 1M-duo v3.0 array

Pal031	Palestine	Human 1M-duo v3.0 array
Pal032	Palestine	Human 1M-duo v3.0 array
Pal033	Palestine	Human 1M-duo v3.0 array
Pal034	Palestine	Human 1M-duo v3.0 array
Pal035	Palestine	Human 1M-duo v3.0 array
Pal036	Palestine	Human 1M-duo v3.0 array
Pal037	Palestine	Human 1M-duo v3.0 array
Pal038	Palestine	Human 1M-duo v3.0 array
Pal039	Palestine	Human 1M-duo v3.0 array
Pal040	Palestine	Human 1M-duo v3.0 array
Pal041	Palestine	Human 1M-duo v3.0 array
Qa001	Qatar	Human 1M-duo v3.0 array
Qa002	Qatar	Human 1M-duo v3.0 array
Qa003	Qatar	Human 1M-duo v3.0 array
Qa004	Qatar	Human 1M-duo v3.0 array
Qa005	Qatar	Human 1M-duo v3.0 array
Qa006	Qatar	Human 1M-duo v3.0 array
Qa007	Qatar	Human 1M-duo v3.0 array
Qa008	Qatar	Human 1M-duo v3.0 array
Qa009	Qatar	Human 1M-duo v3.0 array
Qa010	Qatar	Human 1M-duo v3.0 array
Qa011	Qatar	Human 1M-duo v3.0 array
Qa012	Qatar	Human 1M-duo v3.0 array
Qa013	Qatar	Human 1M-duo v3.0 array
Qa014	Qatar	Human 1M-duo v3.0 array
Qa015	Qatar	Human 1M-duo v3.0 array
Qa016	Qatar	Human 1M-duo v3.0 array
Qa017	Qatar	Human 1M-duo v3.0 array
Qa018	Qatar	Human 1M-duo v3.0 array
Qa019	Qatar	Human 1M-duo v3.0 array
Qa020	Qatar	Human 1M-duo v3.0 array
Qa021	Qatar	Human 1M-duo v3.0 array
Qa022	Qatar	Human 1M-duo v3.0 array
Qa023	Qatar	Human 1M-duo v3.0 array
Qa024	Qatar	Human 1M-duo v3.0 array
Qa025	Qatar	Human 1M-duo v3.0 array
Qa026	Qatar	Human 1M-duo v3.0 array
Qa027	Qatar	Human 1M-duo v3.0 array
Qa028	Qatar	Human 1M-duo v3.0 array
Qa029	Qatar	Human 1M-duo v3.0 array
Qa030	Qatar	Human 1M-duo v3.0 array
Qa031	Qatar	Human 1M-duo v3.0 array
Qa032	Qatar	Human 1M-duo v3.0 array
Qa033	Qatar	Human 1M-duo v3.0 array
Qa034	Qatar	Human 1M-duo v3.0 array
Qa035	Qatar	Human 1M-duo v3.0 array
Qa036	Qatar	Human 1M-duo v3.0 array
Qa037	Qatar	Human 1M-duo v3.0 array
Qa038	Qatar	Human 1M-duo v3.0 array
Qa039	Qatar	Human 1M-duo v3.0 array

Qa040	Qatar	Human 1M-duo v3.0 array
Qa041	Qatar	Human 1M-duo v3.0 array
Qa042	Qatar	Human 1M-duo v3.0 array
Qa043	Qatar	Human 1M-duo v3.0 array
Qa044	Qatar	Human 1M-duo v3.0 array
Qa045	Qatar	Human 1M-duo v3.0 array
Qa046	Qatar	Human 1M-duo v3.0 array
Qa047	Qatar	Human 1M-duo v3.0 array
Qa048	Qatar	Human 1M-duo v3.0 array
Qa049	Qatar	Human 1M-duo v3.0 array
Qa050	Qatar	Human 1M-duo v3.0 array
Qa051	Qatar	Human 1M-duo v3.0 array
Qa052	Qatar	Human 1M-duo v3.0 array
Qa053	Qatar	Human 1M-duo v3.0 array
Qa054	Qatar	Human 1M-duo v3.0 array
Qa055	Qatar	Human 1M-duo v3.0 array
Qa056	Qatar	Human 1M-duo v3.0 array
Qa057	Qatar	Human 1M-duo v3.0 array
Qa058	Qatar	Human 1M-duo v3.0 array
Qa059	Qatar	Human 1M-duo v3.0 array
Qa060	Qatar	Human 1M-duo v3.0 array
Qa061	Qatar	Human 1M-duo v3.0 array
Qa062	Qatar	Human 1M-duo v3.0 array
Qa063	Qatar	Human 1M-duo v3.0 array
Qa064	Qatar	Human 1M-duo v3.0 array
Qa065	Qatar	Human 1M-duo v3.0 array
Qa066	Qatar	Human 1M-duo v3.0 array
Qa067	Qatar	Human 1M-duo v3.0 array
Qa068	Qatar	Human 1M-duo v3.0 array
Qa069	Qatar	Human 1M-duo v3.0 array
Qa070	Qatar	Human 1M-duo v3.0 array
Qa071	Qatar	Human 1M-duo v3.0 array
Qa072	Qatar	Human 1M-duo v3.0 array
Qa073	Qatar	Human 1M-duo v3.0 array
Qa074	Qatar	Human 1M-duo v3.0 array
Qa075	Qatar	Human 1M-duo v3.0 array
Qa076	Qatar	Human 1M-duo v3.0 array
Qa077	Qatar	Human 1M-duo v3.0 array
Qa078	Qatar	Human 1M-duo v3.0 array
Qa079	Qatar	Human 1M-duo v3.0 array
Qa080	Qatar	Human 1M-duo v3.0 array
Qa081	Qatar	Human 1M-duo v3.0 array
Qa082	Qatar	Human 1M-duo v3.0 array
Qa083	Qatar	Human 1M-duo v3.0 array
Qa084	Qatar	Human 1M-duo v3.0 array
Qa085	Qatar	Human 1M-duo v3.0 array
Qa086	Qatar	Human 1M-duo v3.0 array
Qa087	Qatar	Human 1M-duo v3.0 array
Qa088	Qatar	Human 1M-duo v3.0 array
Qa089	Qatar	Human 1M-duo v3.0 array

Qa090	Qatar	Human 1M-duo v3.0 array
Qa091	Qatar	Human 1M-duo v3.0 array
Qa092	Qatar	Human 1M-duo v3.0 array
Qa093	Qatar	Human 1M-duo v3.0 array
Qa094	Qatar	Human 1M-duo v3.0 array
Qa095	Qatar	Human 1M-duo v3.0 array
Qa096	Qatar	Human 1M-duo v3.0 array
Qa097	Qatar	Human 1M-duo v3.0 array
Qa098	Qatar	Human 1M-duo v3.0 array
Qa099	Qatar	Human 1M-duo v3.0 array
Qa100	Qatar	Human 1M-duo v3.0 array
Qa101	Qatar	Human 1M-duo v3.0 array
Qa102	Qatar	Human 1M-duo v3.0 array
Qa103	Qatar	Human 1M-duo v3.0 array
Qa104	Qatar	Human 1M-duo v3.0 array
Qa105	Qatar	Human 1M-duo v3.0 array
Qa106	Qatar	Human 1M-duo v3.0 array
Qa107	Qatar	Human 1M-duo v3.0 array
Qa108	Qatar	Human 1M-duo v3.0 array
Qa109	Qatar	Human 1M-duo v3.0 array
Qa110	Qatar	Human 1M-duo v3.0 array
Qa111	Qatar	Human 1M-duo v3.0 array
Qa112	Qatar	Human 1M-duo v3.0 array
Qa113	Qatar	Human 1M-duo v3.0 array
Qa114	Qatar	Human 1M-duo v3.0 array
Qa115	Qatar	Human 1M-duo v3.0 array
Qa116	Qatar	Human 1M-duo v3.0 array
Qa117	Qatar	Human 1M-duo v3.0 array
Qa118	Qatar	Human 1M-duo v3.0 array
Qa119	Qatar	Human 1M-duo v3.0 array
Qa120	Qatar	Human 1M-duo v3.0 array
Qa121	Qatar	Human 1M-duo v3.0 array
Qa122	Qatar	Human 1M-duo v3.0 array
Qa123	Qatar	Human 1M-duo v3.0 array
Qa124	Qatar	Human 1M-duo v3.0 array
Qa125	Qatar	Human 1M-duo v3.0 array
Qa126	Qatar	Human 1M-duo v3.0 array
Qa127	Qatar	Human 1M-duo v3.0 array
Qa128	Qatar	Human 1M-duo v3.0 array
Qa129	Qatar	Human 1M-duo v3.0 array
Qa130	Qatar	Human 1M-duo v3.0 array
Qa131	Qatar	Human 1M-duo v3.0 array
KSA001	KSA	Human 1M-duo v3.0 array
KSA002	KSA	Human 1M-duo v3.0 array
KSA003	KSA	Human 1M-duo v3.0 array
KSA004	KSA	Human 1M-duo v3.0 array
KSA005	KSA	Human 1M-duo v3.0 array
KSA006	KSA	Human 1M-duo v3.0 array
KSA007	KSA	Human 1M-duo v3.0 array
KSA008	KSA	Human 1M-duo v3.0 array

KSA009	KSA	Human 1M-duo v3.0 array
So001	Somalia	Human 1M-duo v3.0 array
So002	Somalia	Human 1M-duo v3.0 array
So003	Somalia	Human 1M-duo v3.0 array
So004	Somalia	Human 1M-duo v3.0 array
So005	Somalia	Human 1M-duo v3.0 array
So006	Somalia	Human 1M-duo v3.0 array
Su001	Sudan	Human 1M-duo v3.0 array
Su002	Sudan	Human 1M-duo v3.0 array
Su003	Sudan	Human 1M-duo v3.0 array
Su004	Sudan	Human 1M-duo v3.0 array
Su005	Sudan	Human 1M-duo v3.0 array
Su006	Sudan	Human 1M-duo v3.0 array
Su007	Sudan	Human 1M-duo v3.0 array
Su008	Sudan	Human 1M-duo v3.0 array
Su009	Sudan	Human 1M-duo v3.0 array
Su010	Sudan	Human 1M-duo v3.0 array
Su011	Sudan	Human 1M-duo v3.0 array
Su012	Sudan	Human 1M-duo v3.0 array
Su013	Sudan	Human 1M-duo v3.0 array
Su014	Sudan	Human 1M-duo v3.0 array
Su015	Sudan	Human 1M-duo v3.0 array
Su016	Sudan	Human 1M-duo v3.0 array
Su017	Sudan	Human 1M-duo v3.0 array
Su018	Sudan	Human 1M-duo v3.0 array
Su019	Sudan	Human 1M-duo v3.0 array
Su020	Sudan	Human 1M-duo v3.0 array
Su021	Sudan	Human 1M-duo v3.0 array
Su022	Sudan	Human 1M-duo v3.0 array
Su023	Sudan	Human 1M-duo v3.0 array
Su024	Sudan	Human 1M-duo v3.0 array
Su025	Sudan	Human 1M-duo v3.0 array
Su026	Sudan	Human 1M-duo v3.0 array
Su027	Sudan	Human 1M-duo v3.0 array
Su028	Sudan	Human 1M-duo v3.0 array
Sy001	Syria	Human 1M-duo v3.0 array
Sy002	Syria	Human 1M-duo v3.0 array
Sy003	Syria	Human 1M-duo v3.0 array
Sy004	Syria	Human 1M-duo v3.0 array
Sy005	Syria	Human 1M-duo v3.0 array
Sy006	Syria	Human 1M-duo v3.0 array
Tu001	Tunisia	Human 1M-duo v3.0 array
Tu002	Tunisia	Human 1M-duo v3.0 array
Tu003	Tunisia	Human 1M-duo v3.0 array
Tu004	Tunisia	Human 1M-duo v3.0 array
Tu005	Tunisia	Human 1M-duo v3.0 array
Tu006	Tunisia	Human 1M-duo v3.0 array
Tu007	Tunisia	Human 1M-duo v3.0 array
Tu008	Tunisia	Human 1M-duo v3.0 array
Tu009	Tunisia	Human 1M-duo v3.0 array

Ye001	Yemen	Human 1M-duo v3.0 array
Ye002	Yemen	Human 1M-duo v3.0 array
Ye003	Yemen	Human 1M-duo v3.0 array
Ye004	Yemen	Human 1M-duo v3.0 array
Ye005	Yemen	Human 1M-duo v3.0 array
Ye006	Yemen	Human 1M-duo v3.0 array
Ye007	Yemen	Human 1M-duo v3.0 array
Ye008	Yemen	Human 1M-duo v3.0 array
Ye009	Yemen	Human 1M-duo v3.0 array
Ye010	Yemen	Human 1M-duo v3.0 array
Ye011	Yemen	Human 1M-duo v3.0 array
Ye012	Yemen	Human 1M-duo v3.0 array
Ye013	Yemen	Human 1M-duo v3.0 array
Ye014	Yemen	Human 1M-duo v3.0 array
Ye015	Yemen	Human 1M-duo v3.0 array
Ye016	Yemen	Human 1M-duo v3.0 array
Ye017	Yemen	Human 1M-duo v3.0 array
Ye018	Yemen	Human 1M-duo v3.0 array
Ye019	Yemen	Human 1M-duo v3.0 array
Ye020	Yemen	Human 1M-duo v3.0 array
Ye021	Yemen	Human 1M-duo v3.0 array
Ye022	Yemen	Human 1M-duo v3.0 array
Ye023	Yemen	Human 1M-duo v3.0 array
EGYPTLC5514996	Egypt	Illumina Omni 2.5M
EGYPTLC5514997	Egypt	Illumina Omni 2.5M
EGYPTLC5514998	Egypt	Illumina Omni 2.5M
EGYPTLC5514999	Egypt	Illumina Omni 2.5M
EGYPTLC5515000	Egypt	Illumina Omni 2.5M
EGYPTLC5515001	Egypt	Illumina Omni 2.5M
EGYPTLC5515005	Egypt	Illumina Omni 2.5M
EGYPTLC5515006	Egypt	Illumina Omni 2.5M
EGYPTLC5515007	Egypt	Illumina Omni 2.5M
EGYPTLC5515008	Egypt	Illumina Omni 2.5M
EGYPTLC5515009	Egypt	Illumina Omni 2.5M
EGYPTLC5515010	Egypt	Illumina Omni 2.5M
EGYPTLC5515011	Egypt	Illumina Omni 2.5M
EGYPTLC5515013	Egypt	Illumina Omni 2.5M
EGYPTLC5515015	Egypt	Illumina Omni 2.5M
EGYPTLC5515016	Egypt	Illumina Omni 2.5M
EGYPTLC5515017	Egypt	Illumina Omni 2.5M
EGYPTLC5515018	Egypt	Illumina Omni 2.5M
EGYPTLC5515019	Egypt	Illumina Omni 2.5M
EGYPTLC5515020	Egypt	Illumina Omni 2.5M
EGYPTLC5515021	Egypt	Illumina Omni 2.5M
EGYPTLC5515023	Egypt	Illumina Omni 2.5M
EGYPTLC5515027	Egypt	Illumina Omni 2.5M
EGYPTLC5515028	Egypt	Illumina Omni 2.5M
EGYPTLC5515029	Egypt	Illumina Omni 2.5M
EGYPTLC5515031	Egypt	Illumina Omni 2.5M
EGYPTLC5515034	Egypt	Illumina Omni 2.5M

EGYPTLC5515035	Egypt	Illumina Omni 2.5M
EGYPTLC5515036	Egypt	Illumina Omni 2.5M
EGYPTLC5515040	Egypt	Illumina Omni 2.5M
EGYPTLC5515041	Egypt	Illumina Omni 2.5M
EGYPTLC5515042	Egypt	Illumina Omni 2.5M
EGYPTLC5515044	Egypt	Illumina Omni 2.5M
EGYPTLC5515045	Egypt	Illumina Omni 2.5M
EGYPTLC5515046	Egypt	Illumina Omni 2.5M
EGYPTLC5515047	Egypt	Illumina Omni 2.5M
EGYPTLC5515048	Egypt	Illumina Omni 2.5M
EGYPTLC5515052	Egypt	Illumina Omni 2.5M
EGYPTLC5515053	Egypt	Illumina Omni 2.5M
EGYPTLC5515054	Egypt	Illumina Omni 2.5M
EGYPTLC5515061	Egypt	Illumina Omni 2.5M
EGYPTLC5515063	Egypt	Illumina Omni 2.5M
EGYPTLC5515064	Egypt	Illumina Omni 2.5M
EGYPTLC5515066	Egypt	Illumina Omni 2.5M
EGYPTLC5515067	Egypt	Illumina Omni 2.5M
EGYPTLC5515068	Egypt	Illumina Omni 2.5M
EGYPTLC5515069	Egypt	Illumina Omni 2.5M
EGYPTLC5515070	Egypt	Illumina Omni 2.5M
EGYPTLC5515072	Egypt	Illumina Omni 2.5M
EGYPTLC5515075	Egypt	Illumina Omni 2.5M
EGYPTLC5515079	Egypt	Illumina Omni 2.5M
EGYPTLC5515080	Egypt	Illumina Omni 2.5M
EGYPTLC5515082	Egypt	Illumina Omni 2.5M
EGYPTLC5515083	Egypt	Illumina Omni 2.5M
EGYPTLC5515084	Egypt	Illumina Omni 2.5M
EGYPTLC5515085	Egypt	Illumina Omni 2.5M
EGYPTLC5515086	Egypt	Illumina Omni 2.5M
EGYPTLC5515087	Egypt	Illumina Omni 2.5M
EGYPTLC5515088	Egypt	Illumina Omni 2.5M
EGYPTLC5515091	Egypt	Illumina Omni 2.5M
EGYPTLC5515092	Egypt	Illumina Omni 2.5M
EGYPTLC5515093	Egypt	Illumina Omni 2.5M
EGYPTLC5515094	Egypt	Illumina Omni 2.5M
EGYPTLC5515099	Egypt	Illumina Omni 2.5M
EGYPTLC5515100	Egypt	Illumina Omni 2.5M
EGYPTLC5515101	Egypt	Illumina Omni 2.5M
EGYPTLC5515102	Egypt	Illumina Omni 2.5M
EGYPTLC5515104	Egypt	Illumina Omni 2.5M
EGYPTLC5515105	Egypt	Illumina Omni 2.5M
EGYPTLC5515107	Egypt	Illumina Omni 2.5M
EGYPTLC5515108	Egypt	Illumina Omni 2.5M
EGYPTLC5515109	Egypt	Illumina Omni 2.5M
EGYPTLC5515113	Egypt	Illumina Omni 2.5M
EGYPTLC5515114	Egypt	Illumina Omni 2.5M
EGYPTLC5515115	Egypt	Illumina Omni 2.5M
EGYPTLC5515116	Egypt	Illumina Omni 2.5M
EGYPTLC5515117	Egypt	Illumina Omni 2.5M

EGYPTLC5515119	Egypt	Illumina Omni 2.5M
EGYPTLC5515120	Egypt	Illumina Omni 2.5M
EGYPTLC5515121	Egypt	Illumina Omni 2.5M
EGYPTLC5515124	Egypt	Illumina Omni 2.5M
EGYPTLC5515125	Egypt	Illumina Omni 2.5M
EGYPTLC5515126	Egypt	Illumina Omni 2.5M
EGYPTLC5515127	Egypt	Illumina Omni 2.5M
EGYPTLC5515129	Egypt	Illumina Omni 2.5M
EGYPTLC5515130	Egypt	Illumina Omni 2.5M
EGYPTLC5515131	Egypt	Illumina Omni 2.5M
EGYPTLC5515132	Egypt	Illumina Omni 2.5M
EGYPTLC5515134	Egypt	Illumina Omni 2.5M
EGYPTLC5515135	Egypt	Illumina Omni 2.5M
EGYPTLC5515136	Egypt	Illumina Omni 2.5M
EGYPTLC5515137	Egypt	Illumina Omni 2.5M
EGYPTLC5515138	Egypt	Illumina Omni 2.5M
EGYPTLC5515139	Egypt	Illumina Omni 2.5M
EGYPTLC5515140	Egypt	Illumina Omni 2.5M
EGYPTLC5515141	Egypt	Illumina Omni 2.5M
EGYPTLC5515142	Egypt	Illumina Omni 2.5M
EGYPTLC5515146	Egypt	Illumina Omni 2.5M
EGYPTLC5515147	Egypt	Illumina Omni 2.5M
EGYPTLC5515149	Egypt	Illumina Omni 2.5M
EGYPTLC5515150	Egypt	Illumina Omni 2.5M
EGYPTLC5515152	Egypt	Illumina Omni 2.5M
EGYPTLC5515153	Egypt	Illumina Omni 2.5M
EGYPTLC5515156	Egypt	Illumina Omni 2.5M
EGYPTLC5515157	Egypt	Illumina Omni 2.5M
EGYPTLC5515158	Egypt	Illumina Omni 2.5M
EGYPTLC5515160	Egypt	Illumina Omni 2.5M
EGYPTLC5515162	Egypt	Illumina Omni 2.5M
EGYPTLC5515163	Egypt	Illumina Omni 2.5M
EGYPTLC5515164	Egypt	Illumina Omni 2.5M
EGYPTLC5515165	Egypt	Illumina Omni 2.5M
EGYPTLC5515168	Egypt	Illumina Omni 2.5M
EGYPTLC5515170	Egypt	Illumina Omni 2.5M
EGYPTLC5515171	Egypt	Illumina Omni 2.5M
EGYPTLC5515174	Egypt	Illumina Omni 2.5M
EGYPTLC5515175	Egypt	Illumina Omni 2.5M
EGYPTLC5515176	Egypt	Illumina Omni 2.5M
egpg5305762	Ethiopia_AMHARA	Illumina Omni 2.5M
egpg5305763	Ethiopia_AMHARA	Illumina Omni 2.5M
egpg5305764	Ethiopia_AMHARA	Illumina Omni 2.5M
egpg5305765	Ethiopia_AMHARA	Illumina Omni 2.5M
egpg5305770	Ethiopia_AMHARA	Illumina Omni 2.5M
egpg5305771	Ethiopia_AMHARA	Illumina Omni 2.5M
egpg5305772	Ethiopia_AMHARA	Illumina Omni 2.5M
egpg5305773	Ethiopia_AMHARA	Illumina Omni 2.5M
egpg5305778	Ethiopia_AMHARA	Illumina Omni 2.5M
egpg5305780	Ethiopia_AMHARA	Illumina Omni 2.5M

egpg5305781	Ethiopia_AMHARA	Illumina Omni 2.5M
egpg5305786	Ethiopia_AMHARA	Illumina Omni 2.5M
egpg5305787	Ethiopia_AMHARA	Illumina Omni 2.5M
egpg5305788	Ethiopia_AMHARA	Illumina Omni 2.5M
egpg5305789	Ethiopia_AMHARA	Illumina Omni 2.5M
egpg5305794	Ethiopia_AMHARA	Illumina Omni 2.5M
egpg5305795	Ethiopia_AMHARA	Illumina Omni 2.5M
egpg5305796	Ethiopia_AMHARA	Illumina Omni 2.5M
egpg5305797	Ethiopia_AMHARA	Illumina Omni 2.5M
egpg5305803	Ethiopia_AMHARA	Illumina Omni 2.5M
egpg5305804	Ethiopia_AMHARA	Illumina Omni 2.5M
egpg5305805	Ethiopia_AMHARA	Illumina Omni 2.5M
egpg5305810	Ethiopia_AMHARA	Illumina Omni 2.5M
egpg5305811	Ethiopia_AMHARA	Illumina Omni 2.5M
egpg5305812	Ethiopia_AMHARA	Illumina Omni 2.5M
egpg5305813	Ethiopia_AMHARA	Illumina Omni 2.5M
egpg5305818	Ethiopia_AMHARA	Illumina Omni 2.5M
egpg5305819	Ethiopia_AMHARA	Illumina Omni 2.5M
egpg5305820	Ethiopia_AMHARA	Illumina Omni 2.5M
egpg5305821	Ethiopia_AMHARA	Illumina Omni 2.5M
egpg5305826	Ethiopia_AMHARA	Illumina Omni 2.5M
egpg5305827	Ethiopia_AMHARA	Illumina Omni 2.5M
egpg5305828	Ethiopia_AMHARA	Illumina Omni 2.5M
egpg5305829	Ethiopia_AMHARA	Illumina Omni 2.5M
egpg5305834	Ethiopia_AMHARA	Illumina Omni 2.5M
egpg5305835	Ethiopia_AMHARA	Illumina Omni 2.5M
egpg5305836	Ethiopia_AMHARA	Illumina Omni 2.5M
egpg5305842	Ethiopia_AMHARA	Illumina Omni 2.5M
egpg5305843	Ethiopia_AMHARA	Illumina Omni 2.5M
egpg5305845	Ethiopia_AMHARA	Illumina Omni 2.5M
egpg5305850	Ethiopia_AMHARA	Illumina Omni 2.5M
egpg5305851	Ethiopia_AMHARA	Illumina Omni 2.5M
egpg5305852	Ethiopia_AMHARA	Illumina Omni 2.5M
egpg5305853	Ethiopia_AMHARA	Illumina Omni 2.5M
egpg5305767	Ethiopia_GUMUZ	Illumina Omni 2.5M
egpg5305776	Ethiopia_GUMUZ	Illumina Omni 2.5M
egpg5305783	Ethiopia_GUMUZ	Illumina Omni 2.5M
egpg5305792	Ethiopia_GUMUZ	Illumina Omni 2.5M
egpg5305793	Ethiopia_GUMUZ	Illumina Omni 2.5M
egpg5305799	Ethiopia_GUMUZ	Illumina Omni 2.5M
egpg5305800	Ethiopia_GUMUZ	Illumina Omni 2.5M
egpg5305806	Ethiopia_GUMUZ	Illumina Omni 2.5M
egpg5305807	Ethiopia_GUMUZ	Illumina Omni 2.5M
egpg5305808	Ethiopia_GUMUZ	Illumina Omni 2.5M
egpg5305809	Ethiopia_GUMUZ	Illumina Omni 2.5M
egpg5305814	Ethiopia_GUMUZ	Illumina Omni 2.5M
egpg5305815	Ethiopia_GUMUZ	Illumina Omni 2.5M
egpg5305823	Ethiopia_GUMUZ	Illumina Omni 2.5M
egpg5305824	Ethiopia_GUMUZ	Illumina Omni 2.5M
egpg5305825	Ethiopia_GUMUZ	Illumina Omni 2.5M

egpg5305831	Ethiopia_GUMUZ	Illumina Omni 2.5M
egpg5305838	Ethiopia_GUMUZ	Illumina Omni 2.5M
egpg5305839	Ethiopia_GUMUZ	Illumina Omni 2.5M
egpg5305841	Ethiopia_GUMUZ	Illumina Omni 2.5M
egpg5305846	Ethiopia_GUMUZ	Illumina Omni 2.5M
egpg5305854	Ethiopia_GUMUZ	Illumina Omni 2.5M
egpg5305855	Ethiopia_GUMUZ	Illumina Omni 2.5M
egpg5305857	Ethiopia_GUMUZ	Illumina Omni 2.5M
egpg5305858	Ethiopia_GUMUZ	Illumina Omni 2.5M
egpg5305866	Ethiopia_GUMUZ	Illumina Omni 2.5M
egpg5305874	Ethiopia_GUMUZ	Illumina Omni 2.5M
egpg5305862	Ethiopia_OROMO	Illumina Omni 2.5M
egpg5305864	Ethiopia_OROMO	Illumina Omni 2.5M
egpg5305870	Ethiopia_OROMO	Illumina Omni 2.5M
egpg5305871	Ethiopia_OROMO	Illumina Omni 2.5M
egpg5305872	Ethiopia_OROMO	Illumina Omni 2.5M
egpg5305878	Ethiopia_OROMO	Illumina Omni 2.5M
egpg5305880	Ethiopia_OROMO	Illumina Omni 2.5M
egpg5305886	Ethiopia_OROMO	Illumina Omni 2.5M
egpg5305888	Ethiopia_OROMO	Illumina Omni 2.5M
egpg5305893	Ethiopia_OROMO	Illumina Omni 2.5M
egpg5305894	Ethiopia_OROMO	Illumina Omni 2.5M
egpg5305895	Ethiopia_OROMO	Illumina Omni 2.5M
egpg5305896	Ethiopia_OROMO	Illumina Omni 2.5M
egpg5305903	Ethiopia_OROMO	Illumina Omni 2.5M
egpg5305904	Ethiopia_OROMO	Illumina Omni 2.5M
egpg5305910	Ethiopia_OROMO	Illumina Omni 2.5M
egpg5305919	Ethiopia_OROMO	Illumina Omni 2.5M
egpg5305927	Ethiopia_OROMO	Illumina Omni 2.5M
egpg5305934	Ethiopia_OROMO	Illumina Omni 2.5M
egpg5305941	Ethiopia_OROMO	Illumina Omni 2.5M
egpg5305942	Ethiopia_OROMO	Illumina Omni 2.5M
egpg5305943	Ethiopia_OROMO	Illumina Omni 2.5M
egpg5305949	Ethiopia_OROMO	Illumina Omni 2.5M
egpg5305950	Ethiopia_OROMO	Illumina Omni 2.5M
egpg5305951	Ethiopia_OROMO	Illumina Omni 2.5M
egpg5305873	Ethiopia_SOMALI	Illumina Omni 2.5M
egpg5305881	Ethiopia_SOMALI	Illumina Omni 2.5M
egpg5305889	Ethiopia_SOMALI	Illumina Omni 2.5M
egpg5305913	Ethiopia_SOMALI	Illumina Omni 2.5M
egpg5305920	Ethiopia_SOMALI	Illumina Omni 2.5M
egpg5305921	Ethiopia_SOMALI	Illumina Omni 2.5M
egpg5305929	Ethiopia_SOMALI	Illumina Omni 2.5M
egpg5305936	Ethiopia_SOMALI	Illumina Omni 2.5M
egpg5305937	Ethiopia_SOMALI	Illumina Omni 2.5M
egpg5305944	Ethiopia_SOMALI	Illumina Omni 2.5M
egpg5305945	Ethiopia_SOMALI	Illumina Omni 2.5M
egpg5305952	Ethiopia_SOMALI	Illumina Omni 2.5M
egpg5305954	Ethiopia_SOMALI	Illumina Omni 2.5M
egpg5305955	Ethiopia_SOMALI	Illumina Omni 2.5M

egpg5305957	Ethiopia_SOMALI	Illumina Omni 2.5M
egpg5305964	Ethiopia_SOMALI	Illumina Omni 2.5M
egpg5305970	Ethiopia_SOMALI	Illumina Omni 2.5M
egpg5305971	Ethiopia_SOMALI	Illumina Omni 2.5M
egpg5305972	Ethiopia_SOMALI	Illumina Omni 2.5M
egpg5305973	Ethiopia_SOMALI	Illumina Omni 2.5M
egpg5305979	Ethiopia_SOMALI	Illumina Omni 2.5M
egpg5305980	Ethiopia_SOMALI	Illumina Omni 2.5M
egpg5305981	Ethiopia_SOMALI	Illumina Omni 2.5M
egpg5305986	Ethiopia_SOMALI	Illumina Omni 2.5M
egpg5305987	Ethiopia_SOMALI	Illumina Omni 2.5M
egpg5305988	Ethiopia_SOMALI	Illumina Omni 2.5M
egpg5305989	Ethiopia_SOMALI	Illumina Omni 2.5M
egpg5305997	Ethiopia_SOMALI	Illumina Omni 2.5M
egpg5306002	Ethiopia_SOMALI	Illumina Omni 2.5M
egpg5306003	Ethiopia_SOMALI	Illumina Omni 2.5M
egpg5306004	Ethiopia_SOMALI	Illumina Omni 2.5M
egpg5306005	Ethiopia_SOMALI	Illumina Omni 2.5M
egpg5306010	Ethiopia_SOMALI	Illumina Omni 2.5M
egpg5306011	Ethiopia_SOMALI	Illumina Omni 2.5M
egpg5306012	Ethiopia_SOMALI	Illumina Omni 2.5M
egpg5306018	Ethiopia_SOMALI	Illumina Omni 2.5M
egpg5306019	Ethiopia_SOMALI	Illumina Omni 2.5M
egpg5306020	Ethiopia_SOMALI	Illumina Omni 2.5M
egpg5306026	Ethiopia_SOMALI	Illumina Omni 2.5M
egpg5306034	Ethiopia_SOMALI	Illumina Omni 2.5M
egpg5306036	Ethiopia_SOMALI	Illumina Omni 2.5M
egpg5306042	Ethiopia_SOMALI	Illumina Omni 2.5M
egpg5306043	Ethiopia_SOMALI	Illumina Omni 2.5M
egpg5306044	Ethiopia_SOMALI	Illumina Omni 2.5M
egpg5305959	Ethiopia_WOLAYTA	Illumina Omni 2.5M
egpg5305960	Ethiopia_WOLAYTA	Illumina Omni 2.5M
egpg5305961	Ethiopia_WOLAYTA	Illumina Omni 2.5M
egpg5305967	Ethiopia_WOLAYTA	Illumina Omni 2.5M
egpg5305968	Ethiopia_WOLAYTA	Illumina Omni 2.5M
egpg5305969	Ethiopia_WOLAYTA	Illumina Omni 2.5M
egpg5305974	Ethiopia_WOLAYTA	Illumina Omni 2.5M
egpg5305976	Ethiopia_WOLAYTA	Illumina Omni 2.5M
egpg5305984	Ethiopia_WOLAYTA	Illumina Omni 2.5M
egpg5305991	Ethiopia_WOLAYTA	Illumina Omni 2.5M
egpg5305992	Ethiopia_WOLAYTA	Illumina Omni 2.5M
egpg5305998	Ethiopia_WOLAYTA	Illumina Omni 2.5M
egpg5306000	Ethiopia_WOLAYTA	Illumina Omni 2.5M
egpg5306006	Ethiopia_WOLAYTA	Illumina Omni 2.5M
egpg5306007	Ethiopia_WOLAYTA	Illumina Omni 2.5M
egpg5306008	Ethiopia_WOLAYTA	Illumina Omni 2.5M
egpg5306014	Ethiopia_WOLAYTA	Illumina Omni 2.5M
egpg5306015	Ethiopia_WOLAYTA	Illumina Omni 2.5M
egpg5306016	Ethiopia_WOLAYTA	Illumina Omni 2.5M
egpg5306031	Ethiopia_WOLAYTA	Illumina Omni 2.5M

egpg5306032	Ethiopia_WOLAYTA	Illumina Omni 2.5M
egpg5306038	Ethiopia_WOLAYTA	Illumina Omni 2.5M
egpg5306039	Ethiopia_WOLAYTA	Illumina Omni 2.5M
egpg5306040	Ethiopia_WOLAYTA	Illumina Omni 2.5M
egpg5306045	Ethiopia_WOLAYTA	Illumina Omni 2.5M
egpg5306046	Ethiopia_WOLAYTA	Illumina Omni 2.5M
egpg5306047	Ethiopia_WOLAYTA	Illumina Omni 2.5M
egpg5306048	Ethiopia_WOLAYTA	Illumina Omni 2.5M
3577STDY6068373	Yemen	Illumina Omni 2.5M
3577STDY6068383	Yemen	Illumina Omni 2.5M
3577STDY6068415	Yemen	Illumina Omni 2.5M
3577STDY6068457	Yemen	Illumina Omni 2.5M
3577STDY6068460	Yemen	Illumina Omni 2.5M
3577STDY6068468	Yemen	Illumina Omni 2.5M
3577STDY6068472	Yemen	Illumina Omni 2.5M
3577STDY6068476	Yemen	Illumina Omni 2.5M
3577STDY6068480	Yemen	Illumina Omni 2.5M
3577STDY6068488	Yemen	Illumina Omni 2.5M
3577STDY6068496	Yemen	Illumina Omni 2.5M
3577STDY6068504	Yemen	Illumina Omni 2.5M
3577STDY6068512	Yemen	Illumina Omni 2.5M
3577STDY6068520	Yemen	Illumina Omni 2.5M
3577STDY6068527	Yemen	Illumina Omni 2.5M
3577STDY6068531	Yemen	Illumina Omni 2.5M
3577STDY6068535	Yemen	Illumina Omni 2.5M
3577STDY6068536	Yemen	Illumina Omni 2.5M
3577STDY6068543	Yemen	Illumina Omni 2.5M
3577STDY6068552	Yemen	Illumina Omni 2.5M
R02C02	Koura	Infinium Omni Express-24 v1.2 Bead Chip
R04C01	Koura	Infinium Omni Express-24 v1.2 Bead Chip
R03C01	Koura	Infinium Omni Express-24 v1.2 Bead Chip
R06C01	Koura	Infinium Omni Express-24 v1.2 Bead Chip
R01C02	Koura	Infinium Omni Express-24 v1.2 Bead Chip
R03C01	Koura	Infinium Omni Express-24 v1.2 Bead Chip
R04C02	Koura	Infinium Omni Express-24 v1.2 Bead Chip
R01C01	Koura	Infinium Omni Express-24 v1.2 Bead Chip
R06C02	Koura	Infinium Omni Express-24 v1.2 Bead Chip
R02C02	Koura	Infinium Omni Express-24 v1.2 Bead Chip
R02C01	Koura	Infinium Omni Express-24 v1.2 Bead Chip
R01C02	Koura	Infinium Omni Express-24 v1.2 Bead Chip
R04C01	Koura	Infinium Omni Express-24 v1.2 Bead Chip
R06C02	Koura	Infinium Omni Express-24 v1.2 Bead Chip
