# Supplementary material for: Anatolian genetic ancestry in North Lebanese populations
Source: Sci Rep. 2024 Jul 5;14:15518. doi: 10.1038/s41598-024-66191-x (PMC11226446; doi:10.1038/s41598-024-66191-x)
Supplement: Supplementary file 4 — Supplementary Table S3. [file 41598_2024_66191_MOESM4_ESM.pdf]

Sample	Population	Reference
CRE_AN_18	Cretan	Reitsema
CRE_AV_28	Cretan	Reitsema
CRE_IER_20	Cretan	Reitsema
CRE_KAN_18	Cretan	Reitsema
CRE_KIS_27	Cretan	Reitsema
CRE_MOI_17	Cretan	Reitsema
CRE_NE_11	Cretan	Reitsema
CRE_SIT_4	Cretan	Reitsema
CRE_SIT_8	Cretan	Reitsema
CRE_TZ_10	Cretan	Reitsema
CRE_VIA_25	Cretan	Reitsema
CYP15	Cyprus	Reitsema
CYP19	Cyprus	Reitsema
CYP20	Cyprus	Reitsema
CYP22	Cyprus	Reitsema
CYP25	Cyprus	Reitsema
CYP27	Cyprus	Reitsema
CYP30	Cyprus	Reitsema
CYP32	Cyprus	Reitsema
CYP34	Cyprus	Reitsema
CYP35	Cyprus	Reitsema
CYP37	Cyprus	Reitsema
CYP39	Cyprus	Reitsema
CYP41	Cyprus	Reitsema
CYP42	Cyprus	Reitsema
CYP45	Cyprus	Reitsema
CYP46	Cyprus	Reitsema
DOD1	Dodecanese	Reitsema
DOD2	Dodecanese	Reitsema
DOD3	Dodecanese	Reitsema
DOD4	Dodecanese	Reitsema
DOD5	Dodecanese	Reitsema
DOD6	Dodecanese	Reitsema
DOD7	Dodecanese	Reitsema
664	Dodecanese2	Drineas
665	Dodecanese2	Drineas
666	Dodecanese2	Drineas
667	Dodecanese2	Drineas
668	Dodecanese2	Drineas
669	Dodecanese2	Drineas
670	Dodecanese2	Drineas
671	Dodecanese2	Drineas
672	Dodecanese2	Drineas
673	Dodecanese2	Drineas
AHA15	Greek	Reitsema
ARG1	Greek	Reitsema
ARK3	Greek	Reitsema
ARK19	Greek	Reitsema
CYCL1	Greek	Reitsema

CYCL2	Greek	Reitsema
CYCL3	Greek	Reitsema
CYCL12	Greek	Reitsema
CYCL16	Greek	Reitsema
CYCL19	Greek	Reitsema
CYCL22	Greek	Reitsema
CYCL23	Greek	Reitsema
IL4	Greek	Reitsema
IL21	Greek	Reitsema
KOR42	Greek	Reitsema
LAK8	Greek	Reitsema
MAK103	Greek	Reitsema
MAK118	Greek	Reitsema
MAK127	Greek	Reitsema
MAK147	Greek	Reitsema
MAK175	Greek	Reitsema
MAK196	Greek	Reitsema
MAK249	Greek	Reitsema
MAK337	Greek	Reitsema
MES19	Greek	Reitsema
MES32	Greek	Reitsema
R01C02_738243	Zgharta_District	Current work
R02C02_738243	Zgharta_District	Current work
R03C02_738243	Zgharta_District	Current work
R04C01_738243	Zgharta_District	Current work
R04C02_738243	Zgharta_District	Current work
R05C01_738243	Zgharta_District	Current work
R05C02_738243	Zgharta_District	Current work
R06C01_738243	Zgharta_District	Current work
R06C02_738243	Zgharta_District	Current work
R03C02_832782	Zgharta_District	Current work
R04C02_832782	Zgharta_District	Current work
R05C02_832782	Zgharta_District	Current work
R06C02_832782	Zgharta_District	Current work
R01C01_832782	Zgharta_District	Current work
R01C02_832782	Zgharta_District	Current work
R02C02_832782	Zgharta_District	Current work
R03C02_832782	Zgharta_District	Current work
R04C01_832782	Zgharta_District	Current work
R04C02_832782	Zgharta_District	Current work
R05C01_832782	Zgharta_District	Current work
R05C02_832782	Zgharta_District	Current work
R06C01_832782	Zgharta_District	Current work
R06C02_832782	Zgharta_District	Current work
R01C01_832782	Zgharta_District	Current work
R01C02_832782	Zgharta_District	Current work
R02C01_832782	Zgharta_District	Current work
R02C02_832782	Zgharta_District	Current work
R03C01_832782	Zgharta_District	Current work
R03C02_832782	Zgharta_District	Current work

R04C01_832782	Zgharta_District	Current work
R04C02_832782	Zgharta_District	Current work
R05C01_832782	Zgharta_District	Current work
R05C02_832782	Zgharta_District	Current work
R06C01_832782	Zgharta_District	Current work
R06C02_832782	Zgharta_District	Current work
R01C01_832782	Zgharta_District	Current work
R01C02_832782	Zgharta_District	Current work
R02C01_832782	Zgharta_District	Current work
R02C02_832782	Zgharta_District	Current work
R03C01_832782	Zgharta_District	Current work
R03C02_832782	Zgharta_District	Current work
R04C01_832782	Zgharta_District	Current work
R04C02_832782	Zgharta_District	Current work
R05C01_832782	Zgharta_District	Current work
R05C02_832782	Zgharta_District	Current work
R06C02_832782	Zgharta_District	Current work
R01C01_923644	Zgharta_District	Current work
R01C02_923644	Zgharta_District	Current work
R02C02_923644	Zgharta_District	Current work
R03C01_923644	Zgharta_District	Current work
R03C02_923644	Zgharta_District	Current work
R04C01_923644	Zgharta_District	Current work
R04C02_923644	Zgharta_District	Current work
R05C01_923644	Zgharta_District	Current work
R05C02_923644	Zgharta_District	Current work
R06C01_923644	Zgharta_District	Current work
R01C02_923644	Zgharta_District	Current work
R03C01_923644	Zgharta_District	Current work
R03C02_923644	Zgharta_District	Current work
R04C02_923644	Zgharta_District	Current work
R05C01_923644	Zgharta_District	Current work
R05C02_923644	Zgharta_District	Current work
R06C02_923644	Zgharta_District	Current work
R04C01_923644	Zgharta_District	Current work
R01C01_923644	Zgharta_District	Current work
R01C02_923644	Zgharta_District	Current work
R02C01_923644	Zgharta_District	Current work
R02C02_923644	Zgharta_District	Current work
R03C01_923644	Zgharta_District	Current work
R03C02_923644	Zgharta_District	Current work
R04C02_923644	Zgharta_District	Current work
R05C01_923644	Zgharta_District	Current work
R05C02_923644	Zgharta_District	Current work
R06C01_923644	Zgharta_District	Current work
R06C02_923644	Zgharta_District	Current work
R04C02_923644	Zgharta_District	Current work
R01C01_923644	Zgharta_District	Current work
R05C02_923644	Zgharta_District	Current work
R01C01_923834	Zgharta_District	Current work

R01C02_923834	Zgharta_District	Current work
R02C02_923834	Zgharta_District	Current work
R03C02_923834	Zgharta_District	Current work
R04C01_923834	Zgharta_District	Current work
R04C02_923834	Zgharta_District	Current work
R06C02_923834	Zgharta_District	Current work
R06C02_923834	Zgharta_District	Current work
R01C01_923834	Zgharta_District	Current work
R01C02_923834	Zgharta_District	Current work
R02C01_923834	Zgharta_District	Current work
R02C02_923834	Zgharta_District	Current work
R03C01_923834	Zgharta_District	Current work
R03C02_923834	Zgharta_District	Current work
R04C01_923834	Zgharta_District	Current work
R05C02_923834	Zgharta_District	Current work
R04C02_923834	Zgharta_District	Current work
R05C01_923834	Zgharta_District	Current work
R05C02_923834	Zgharta_District	Current work
R01C01_923834	Zgharta_District	Current work
R01C02_923834	Zgharta_District	Current work
R02C01_923834	Zgharta_District	Current work
R02C02_923834	Zgharta_District	Current work
R03C01_923834	Zgharta_District	Current work
R05C01_923834	Zgharta_District	Current work
R05C02_923834	Zgharta_District	Current work
R02C02_923834	Zgharta_District	Current work
R03C02_923834	Zgharta_District	Current work
R04C02_923834	Zgharta_District	Current work
R05C02_923834	Zgharta_District	Current work
R06C02_923834	Zgharta_District	Current work
R01C02_923834	Zgharta_District	Current work
R02C02_923834	Zgharta_District	Current work
R03C01_923834	Zgharta_District	Current work
R04C01_923834	Zgharta_District	Current work
R04C02_923834	Zgharta_District	Current work
R05C01_923834	Zgharta_District	Current work
R05C02_923834	Zgharta_District	Current work
R01C02_923834	Zgharta_District	Current work
R02C01_923834	Zgharta_District	Current work
R03C02_923834	Zgharta_District	Current work
R05C01_923834	Zgharta_District	Current work
R05C02_923834	Zgharta_District	Current work
R06C02_923834	Zgharta_District	Current work
R02C01_923834	Zgharta_District	Current work
R03C02_923834	Zgharta_District	Current work
R04C01_923834	Zgharta_District	Current work
R05C01_923834	Zgharta_District	Current work
R06C01_923834	Zgharta_District	Current work
R01C01_923834	Zgharta_District	Current work
R01C02_923834	Zgharta_District	Current work

R02C01_923834	Zgharta_District	Current work
R02C02_923834	Zgharta_District	Current work
R03C02_923834	Zgharta_District	Current work
R04C01_923834	Zgharta_District	Current work
R04C02_923834	Zgharta_District	Current work
R05C01_923834	Zgharta_District	Current work
R05C02_923834	Zgharta_District	Current work
R06C01_923834	Zgharta_District	Current work
R06C02_923834	Zgharta_District	Current work
R01C01_923834	Zgharta_District	Current work
R01C02_923834	Zgharta_District	Current work
R02C01_923834	Zgharta_District	Current work
R02C02_923834	Zgharta_District	Current work
R03C01_923834	Zgharta_District	Current work
R03C02_923834	Zgharta_District	Current work
R04C01_923834	Zgharta_District	Current work
R05C01_923834	Zgharta_District	Current work
R06C01_923834	Zgharta_District	Current work
R01C02_923834	Zgharta_District	Current work
R02C01_923834	Zgharta_District	Current work
R02C02_923834	Zgharta_District	Current work
R03C01_923834	Zgharta_District	Current work
R04C02_923834	Zgharta_District	Current work
R06C02_923834	Zgharta_District	Current work
R05C01_923834	Zgharta_District	Current work
R06C01_923834	Zgharta_District	Current work
R02C01_923834	Zgharta_District	Current work
R02C02_923834	Zgharta_District	Current work
R03C02_923834	Zgharta_District	Current work
R04C01_923834	Zgharta_District	Current work
R04C02_923834	Zgharta_District	Current work
R05C01_923834	Zgharta_District	Current work
R06C01_923834	Zgharta_District	Current work
R06C02_923834	Zgharta_District	Current work
R03C02_923834	Zgharta_District	Current work
R04C01_923834	Zgharta_District	Current work
R05C02_923834	Zgharta_District	Current work
R06C01_923834	Zgharta_District	Current work
R05C02_923834	Zgharta_District	Current work
R01C01_923838	Zgharta_District	Current work
R01C02_923838	Zgharta_District	Current work
R02C01_923838	Zgharta_District	Current work
R02C02_923838	Zgharta_District	Current work
R03C01_923838	Zgharta_District	Current work
R04C01_923838	Zgharta_District	Current work
R04C02_923838	Zgharta_District	Current work
R05C01_923838	Zgharta_District	Current work
R05C02_923838	Zgharta_District	Current work
R06C01_923838	Zgharta_District	Current work
R06C02_923838	Zgharta_District	Current work

R01C02_9238388	Zgharta_District	Current work
R06C02_9238388	Zgharta_District	Current work
R02C01_832782	Tripoli_District	Current work
R06C02_923644	Tripoli_District	Current work
R01C01_923644	Tripoli_District	Current work
R06C01_923644	Tripoli_District	Current work
R03C02_923644	Tripoli_District	Current work
R05C01_923644	Tripoli_District	Current work
R06C01_923644	Tripoli_District	Current work
R01C02_923644	Tripoli_District	Current work
R04C01_923644	Tripoli_District	Current work
R06C01_923834	Tripoli_District	Current work
R01C02_923834	Tripoli_District	Current work
R03C02_923834	Tripoli_District	Current work
R05C01_923834	Tripoli_District	Current work
R03C02_923838	Tripoli_District	Current work
R03C01_832782	Mount_Lebanon	Current work
R06C01_832782	Mount_Lebanon	Current work
R05C02_923834	Mount_Lebanon	Current work
R04C02_923644	Koura_District	Current work
R05C02_923644	Koura_District	Current work
R06C01_923644	Koura_District	Current work
R01C01_923644	Koura_District	Current work
R01C02_923644	Koura_District	Current work
R02C01_923644	Koura_District	Current work
R02C02_923644	Koura_District	Current work
R03C01_923644	Koura_District	Current work
R03C02_923644	Koura_District	Current work
R04C01_923644	Koura_District	Current work
R04C02_923644	Koura_District	Current work
R05C01_923644	Koura_District	Current work
R05C02_923644	Koura_District	Current work
R06C02_923644	Koura_District	Current work
R03C01_923644	Koura_District	Current work
R03C02_923644	Koura_District	Current work
R04C02_923644	Koura_District	Current work
R05C01_923644	Koura_District	Current work
R05C02_923644	Koura_District	Current work
R06C01_923644	Koura_District	Current work
R06C02_923644	Koura_District	Current work
R02C01_923644	Koura_District	Current work
R02C02_923644	Koura_District	Current work
R03C01_923644	Koura_District	Current work
R03C02_923644	Koura_District	Current work
R05C01_923644	Koura_District	Current work
R06C01_923644	Koura_District	Current work
R06C02_923644	Koura_District	Current work
R05C02_923834	Koura_District	Current work
R04C02_923834	Koura_District	Current work
R05C02_923834	Koura_District	Current work

R06C02_923834	Koura_District	Current work
R01C01_923834	Koura_District	Current work
R01C02_923834	Koura_District	Current work
R02C01_923834	Koura_District	Current work
R02C02_923834	Koura_District	Current work
R03C02_923834	Koura_District	Current work
R04C01_923834	Koura_District	Current work
R04C02_923834	Koura_District	Current work
R05C02_923834	Koura_District	Current work
R06C01_923834	Koura_District	Current work
R06C02_923834	Koura_District	Current work
R04C02_923834	Koura_District	Current work
R05C02_923834	Koura_District	Current work
R01C01_923834	Koura_District	Current work
R01C02_923834	Koura_District	Current work
R02C02_923834	Koura_District	Current work
R03C01_923834	Koura_District	Current work
R04C02_923834	Koura_District	Current work
R05C02_923834	Koura_District	Current work
R06C02_923834	Koura_District	Current work
R02C01_923834	Koura_District	Current work
R02C02_923834	Koura_District	Current work
R03C01_923834	Koura_District	Current work
R04C01_923834	Koura_District	Current work
R04C02_923834	Koura_District	Current work
R06C02_923834	Koura_District	Current work
R01C01_923838	Koura_District	Current work
R02C01_923838	Koura_District	Current work
R02C02_923838	Koura_District	Current work
R03C02_923838	Koura_District	Current work
R05C02_923838	Koura_District	Current work
R01C01_923838	Koura_District	Current work
R02C01_923644	Dinniyeh_District	Current work
R02C01_923644	Dinniyeh_District	Current work
R02C02_923644	Dinniyeh_District	Current work
R04C01_923644	Dinniyeh_District	Current work
R04C01_923644	Dinniyeh_District	Current work
R03C02_923644	Dinniyeh_District	Current work
R05C02_923644	Dinniyeh_District	Current work
R06C02_923644	Dinniyeh_District	Current work
R03C01_923834	Dinniyeh_District	Current work
R05C01_923834	Dinniyeh_District	Current work
R06C01_923834	Dinniyeh_District	Current work
R04C02_923834	Dinniyeh_District	Current work
R05C01_923834	Dinniyeh_District	Current work
R06C01_923834	Dinniyeh_District	Current work
R06C02_923834	Dinniyeh_District	Current work
R02C02_923834	Dinniyeh_District	Current work
R03C02_923834	Dinniyeh_District	Current work
R06C01_923834	Dinniyeh_District	Current work

R06C02_9238348	Dinniyeh_District	Current work
R03C02_9238348	Dinniyeh_District	Current work
R04C01_9238348	Dinniyeh_District	Current work
R04C02_9238348	Dinniyeh_District	Current work
R06C01_9238348	Dinniyeh_District	Current work
R06C02_9238348	Dinniyeh_District	Current work
R01C01_9238348	Dinniyeh_District	Current work
R02C01_9238348	Dinniyeh_District	Current work
R03C02_9238348	Dinniyeh_District	Current work
R06C01_9238348	Dinniyeh_District	Current work
R06C02_9238348	Dinniyeh_District	Current work
R01C01_9238348	Dinniyeh_District	Current work
R04C01_9238348	Dinniyeh_District	Current work
R04C02_9238348	Dinniyeh_District	Current work
R06C01_9238348	Dinniyeh_District	Current work
R01C01_9238348	Dinniyeh_District	Current work
R01C02_9238348	Dinniyeh_District	Current work
R03C01_9238348	Dinniyeh_District	Current work
R01C01_9238348	Dinniyeh_District	Current work
R03C02_9238348	Dinniyeh_District	Current work
R04C01_9238348	Dinniyeh_District	Current work
R05C01_9238348	Dinniyeh_District	Current work
R05C02_9238348	Dinniyeh_District	Current work
R04C01_9238348	Dinniyeh_District	Current work
R01C01_9238348	Dinniyeh_District	Current work
R01C02_9238348	Dinniyeh_District	Current work
R03C01_9238348	Dinniyeh_District	Current work
R05C02_9238348	Dinniyeh_District	Current work
R01C01_9238349	Dinniyeh_District	Current work
R01C02_9238349	Dinniyeh_District	Current work
R02C01_9238349	Dinniyeh_District	Current work
R02C02_9238349	Dinniyeh_District	Current work
R03C01_9238349	Dinniyeh_District	Current work
R04C02_9238349	Dinniyeh_District	Current work
R05C01_9238349	Dinniyeh_District	Current work
R06C02_9238349	Dinniyeh_District	Current work
R01C01_9238349	Dinniyeh_District	Current work
R02C01_9238348	Dinniyeh_District	Current work
R02C02_9236445	Dinniyeh_District	Current work
